# A Systems Biology Comparison of Ovarian Cancers Implicates Putative Somatic Driver Mutations through Protein-Protein Interaction Models

**DOI:** 10.1371/journal.pone.0163353

**Published:** 2016-10-27

**Authors:** Mary Qu Yang, Laura Elnitski

**Affiliations:** 1 MidSouth Bioinformatics Center and Joint Bioinformatics Ph.D. Program, University of Arkansas at Little Rock and University of Arkansas for Medical Sciences, 2801 S. University Avenue, Little Rock, Arkansas, 72204, United States of America; 2 National Human Genome Research Institute, National Institutes of Health, Rockville, MD, 20852, United States of America; Johns Hopkins University, UNITED STATES

## Abstract

Ovarian carcinomas can be aggressive with a high mortality rate (e.g., high-grade serous ovarian carcinomas, or HGSOCs), or indolent with much better long-term outcomes (e.g., low-malignant-potential, or LMP, serous ovarian carcinomas). By comparing LMP and HGSOC tumors, we can gain insight into the mechanisms underlying malignant progression in ovarian cancer. However, previous studies of the two subtypes have been focused on gene expression analysis. Here, we applied a systems biology approach, integrating gene expression profiles derived from two independent data sets containing both LMP and HGSOC tumors with protein-protein interaction data. Genes and related networks implicated by both data sets involved both known and novel disease mechanisms and highlighted the different roles of *BRCA1* and *CREBBP* in the two tumor types. In addition, the incorporation of somatic mutation data revealed that amplification of *PAK4* is associated with poor survival in patients with HGSOC. Thus, perturbations in protein interaction networks demonstrate differential trafficking of network information between malignant and benign ovarian cancers. The novel network-based molecular signatures identified here may be used to identify new targets for intervention and to improve the treatment of invasive ovarian cancer as well as early diagnosis.

## Introduction

Ovarian cancer is the most lethal gynecological malignancy, and serous carcinomas of the ovary account for the majority of ovarian cancer deaths[1]. Papillary serous ovarian cancer, the most common ovarian tumor subtype, comprises a spectrum of disease, ranging from invasive carcinomas to benign, low-malignant-potential (LMP) tumors. Invasive serous carcinomas have been further subdivided into low-grade and high-grade subtypes based on molecular characteristics, disrupted functional pathways, and patient outcomes[2]. Low-grade serous carcinomas have characteristics similar to those of LMP tumors; both differ substantially from high-grade serous carcinomas (HGSOCs) [2–6]. Currently, it is poorly understood why LMP tumors follow a benign clinical course, despite their malignant features and metastatic potential, whereas HGSOCs are very aggressive and can spread quickly to other organs.

Comparisons of LMP and HGSOC tumors may offer unique insights into malignant ovarian tumors by revealing the characteristics of aggressive tumors. However, the methods available to make such comparisons have significant limitations. Genome-wide microarray data have been used widely in ovarian cancer research [7–13] to provide information on the relative abundance of transcripts [14, 15]. But these expression profiles do not reveal the presence of somatic mutations. By contrast, whole genome sequencing and exome sequencing can be used to identify disease-associated mutations. For example, the Cancer Genome Atlas (TCGA) Consortium recently published a large-scale, comprehensive overview of HGSOCs that revealed recurrent *TP53* mutations in more than 90% of samples [16]. What is currently missing is an integrative means of analysis designed to identify somatic mutations that affect downstream gene expression levels and protein interaction networks. By integrating expression and mutation data, we can better identify driver mutations and dysregulated functional pathways present in cancer cells.

Models depicting protein-protein interaction (PPI) data provide a useful proxy for cellular communication lattices [17, 18] and, when integrated with transcript expression data, can reveal disruptions cascading from gene mutations or other functional alterations. The communication lattice consists of a grid-like matrix of multiple interleaved protein interaction networks, including smaller focal interaction units known as subnetworks. These individual components constitute single targeted outcomes within the larger, more dispersed framework of the network. Tying together the interactions of multiple subnetworks are centralized conduits, or hub proteins, such as MYC, P53, and EGFR, which play information-trafficking roles in the cell. Hub proteins are defined as the proteins with the highest numbers of interactions with other proteins in the proteome [19–21]. Due to their essential functions, the mutation, deletion, or functional alteration of hub proteins causes severe, though rare, phenotypic outcomes [22–24]. Thus, proteins severely affected by mutation are more likely to reside in less central network locations. A network-based method that incorporates transcriptome and human interactome enable the identification of genes acting as regulators by mediating expression of downstream genes and play essential roles in disease.

Here, we integrate the vast amount of expression data available from HGSOC and LMP microarrays with PPI data to identify disrupted downstream PPIs. Our method proceeds by objectively selecting the most strongly affected protein interactions from over 100,000 options. We also demonstrate our method’s predictive ability by using our gene set to classify an independent set of ovarian tumor samples according to HGSOC or LMP subtype. This information may aid diagnosis of HGSOC, which is often asymptomatic in its early stages and thus detected late, contributing to the low survival rate associated with this subtype [7, 25, 26]. In addition to strengthening our ability to classify tumors at the molecular level, this type of systems biology approach aids in identifying molecular perturbations that are camouflaged in gene expression data, providing insight into the biological mechanisms underlying cancer.

## Results

### Comparison of methods to rank genes with respect to differential expression

**Fig 1:**
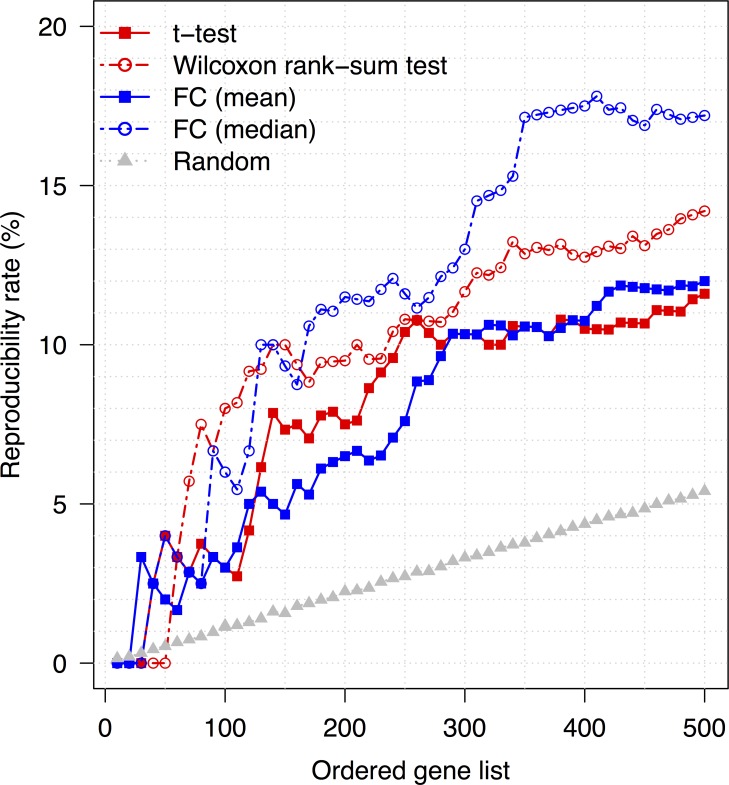
Reproducibility rates for various methods of identifying genes differentially expressed between low-malignant-potential (LMP) and high-grade serous carcinoma (HGSOC) samples in two data sets, GSE17308 (9) and GSE9891 (12). Genes were selected by: the *P*-values from t-tests (*solid red line*); the *P*-values from Wilcoxon rank-sum tests (*dotted red line*); mean fold change (FC; *solid blue line*); median FC (*dotted blue line*); and random selection (*dotted grey line*). Reproducibility was defined as the percentage of differentially expressed genes from one data set’s list also included in the other data set’s list.

Two independent microarray expression profiles, GSE17308 [9] and GSE9891 [12], were obtained from the Gene Expression Omnibus (GEO) to compare LMP and HGSOC samples across independent patient groups. We first created lists of genes ranked by fold-change and statistical significance [27]. We then compared the reproducibility rate of lists created with fold change in mean values, fold change in median values, t-test *P*-values, and Wilcoxon rank-sum test *P*-values, selecting sets of genes at random to calculate the statistical significance of our findings (**Fig 1**). Here, reproducibility was defined as the percentage of differentially expressed genes from one expression profile’s list also included in the other profile’s list (i.e., those genes identified by both the GSE17308 and GSE9891 comparisons). The reproducibility rate of the lists generated with each of the four methods was significantly higher than the rate for the random gene list, as indicated by *P* <2 × 10^−12^ for all four statistical tests. However, the lists compiled on the basis of fold-change in median expression values demonstrated a higher rate of reproducibility than those compiled on the basis of fold-change in mean expression levels (**Fig 1**). Further, we evaluated the effectiveness of these gene lists in discriminating between different phenotypes using hierarchical cluster analysis. More homogeneous sample clusters were achieved with the top-ranked genes from the Wilcoxon rank-sum test (**S1A Fig**) than with those identified by median fold change (**S1B Fig**), with homogeneity being defined as how well the selected genes were able to separate disease types (i.e., LMP vs HGSOC). In other words, more homogeneous clusters contain more samples with the same phenotype. For instance, the bottoms of panels A and B in **S1 Fig** display the hierarchical clusters of samples from GSE17308. Five of seven HGSOC tissue samples were placed in the invasive clusters, indicating less homogeneous clusters, by median fold change, whereas all seven samples were correctly clustered together by the genes from the Wilcoxon rank-sum test.

Thus, to simultaneously maximize reproducibility and effectiveness at classifying tumor types, as well as ensure that differential expression was sufficiently large to be biological meaningful [28], we used both the Wilcoxon rank-sum test and median-fold change to identify differentially expressed genes in subsequent analyses.

### Analysis of differentially expressed genes concordant between expression profiles

#### Differential gene expression

**Fig 2:**
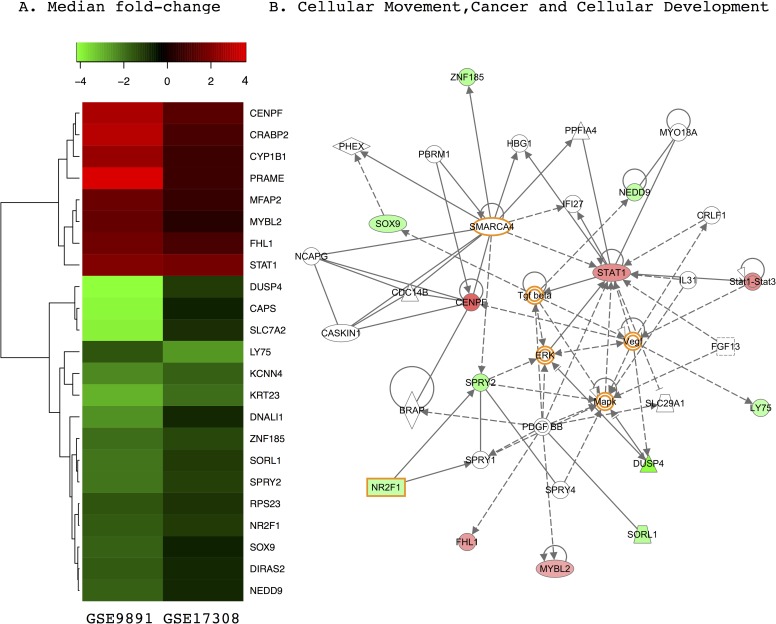
Differentially expressed genes concordant between two data sets containing low-malignant-potential (LMP) and high-grade serous carcinoma (HGSOC) samples. (A) Heatmap of 23 genes differentially expressed between LMP and HGSOC tumors in two independent GEO expression profiles, GSE17308 (9) and GSE9891 (12), organized by median fold change. (B) The “cellular movement, cancer, and cellular development” network is enriched for genes differentially expressed between LMP and HGSOC, as determined by pathway analysis. In both panels, red represents overexpression and green represents underexpression in HGSOC. Genes highlighted in orange are well-known ovarian cancer genes that have been used as biomarkers and targets for drug design.

We identified 195 differentially expressed genes (*P* <0.01, FC >0.6) for GSE17308 and 230 (*P* <10^−6^ and FC >1.5) for GSE9891. To attain similar numbers of top-ranked differentially expressed genes, different thresholds were adopted. The distributions of the median fold-changes and the *P*-values from the Wilcoxon rank-sum test differed between the two GEO data sets (**S2 Fig)** were varied, which may cause by different platforms used in experiments for attaining expression profiles and other technical variations [27]. The list of genes differentially expressed between tumor types in the two independently generated GEO data sets converged on a common set of 23 genes (8 upregulated and 15 downregulated in HGSOC) that were directionally consistent and statistically significant in both expression profiles (**Fig 2A**). To measure the reliability of these data, we used expression levels of these 23 genes to perform hierarchical clustering of a third, independent patient expression profile (GSE27651) [29] containing LMP and HGSOC samples (**S3 Fig**). Not a single one of the tissue samples in this third data set was clustered erroneously.

#### Data interpretation using pathway analysis

Six of the 23 differentially expressed genes have already been implicated in the ovarian cancer literature (*STAT1*, *MYBL2*, *SPRY2*, *NR2F1*, *DUSP4*, and *RPS23*) [30–35]. However, most of these genes represent potentially novel disease biomarkers. An Ingenuity pathway analysis showed that the list of 23 genes is enriched in tumor-related processes such as cellular movement, cancer, and cellular development (12 genes, **Fig 2B**) and cell cycle, drug metabolism, and molecular transport (11 genes, **S4 Fig**). Importantly, *DUSP4*, a known cancer risk gene [36], is represented in both of these pathways, suggesting pleiotropy with multiple functional associations. In addition, the analysis identified canonical networks whose members relay signals from the plasma membrane to the nucleus. Pathways enriched for this gene list included aryl hydrocarbon receptor (AhR) signaling, which is linked to homologous recombination [37]; chromosomal instability, as reported in HGSOC [16]; and retinoic acid receptor (RAR) activation. Thus, LMP and HGSOC tumors exhibit differences in cellular pathways, as revealed by altered expression of the genes involved.

The static mapping of differentially expressed genes onto network and pathway illustrations showed that known disease processes are affected by differential gene expression (**Fig 2B and S4 Fig**). However, contrary to our expectation, the majority of affected genes were localized to the periphery of the pathway/network maps rather than to interior positions, suggesting limited impact on information dissemination. By contrast, some well known ovarian cancer genes whose expression was not altered, such as *VEGF*, *ERK*, *MAPK*, *TGF-β*, and *SMARCA4* [38–45], displayed centralized placement in these same network maps, indicating high connectivity within the gene networks. We postulated that genes encoding proteins with important regulatory functions may not be differentially expressed themselves but could mediate downstream effects on genes whose expression levels were altered in our microarray meta-analysis.

### Subnetwork Analysis

To identify characteristics that can be used to discriminate between HGSOC and LMP samples, we employed a subnetwork analysis method adapted from Chuang et al. [46] and projected pairwise PPI data onto a larger combinatorial lattice informed by gene expression values. We overlaid the expression value measured for each gene onto its corresponding protein, as we built an increasingly large, interconnected protein framework. We searched for subnetwork conduits whose differential activities scored as being highly discriminative for tumor type, as assessed by amutual information score and statistical thresholds (see Materials and Methods). Starting from a single gene as a subnetwork “seed,” neighboring genes were added to expand the interaction framework by prioritizing choices that maximized the differential scores between tumor types; a computationally intensive approach was used to check all options before proceeding to the next addition. To ascertain the statistical significance of the differential networks, we compared our finished network data to a null distribution, which we generated by randomizing tumor type assignments or gene assignments within each subnetwork. We then sampled the randomized sets 10,000 times to evaluate the statistical significance of the results returned for each subnetwork. In this way, we identified 175 subnetworks from the GSE9891 data set and 179 from the GSE17308 data set that were differentially affected in HGSOC and LMP tumors (each with *P* ≤10^−4^, discrimination score, or DS ≥0.66; **S1 Table**). Among these subnetworks, we found 75 genes including 9 seed genes implicated in both data sets.

#### Differential expression and subnetwork participation

Six genes present in the significantly impacted subnetworks also showed differential expression in both data sets: *STAT1*, *MYBL2*, *RPS23*, *NR2F1*, *SOX9*, and *SPRY2*. Each gene participated in from one to eight subnetworks, the components of which showed enrichment in gene ontology (GO) terms for the cell cycle, apoptosis, regulation of transcription, signal processing, cell communication, and receptor protein tyrosine kinase signaling pathways.

#### Differential subnetwork participants associated with tumor type

**Fig 3:**
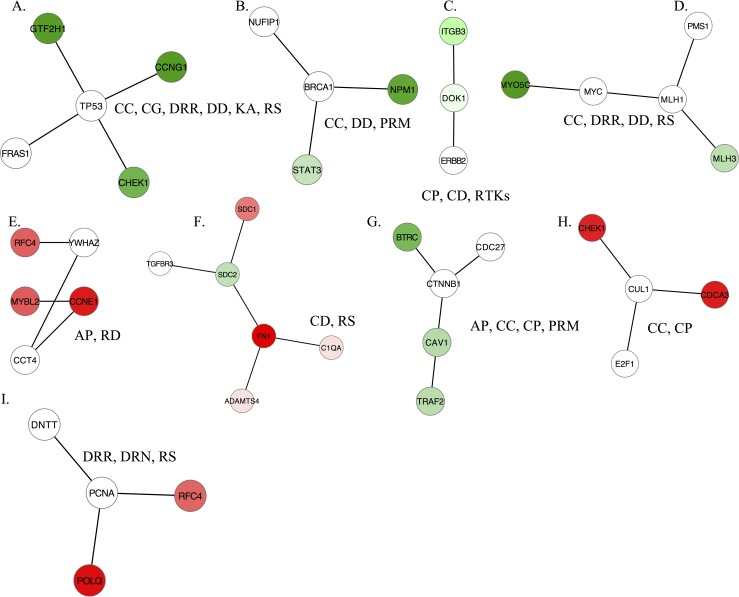
Representative subnetworks that discriminate between low-malignant-potential (LMP) and high-grade serous carcinoma (HGSOC) samples. (A-I) Subnetworks include genes such as *TP53*, *BRCA1*, and *MYC*, which are mutated in ovarian carcinomas although their expression level is not significantly altered (*white nodes*). Red nodes indicate overexpression of genes in HGSOC subnetworks, and green nodes indicate underexpression. Abbreviations: cell cycle (CC), cell growth (CG), cell proliferation (CP), cell differentiation (CD), DNA damage (DD), DNA repair (DDR), DNA replication (DRN), regulation of kinase activity (RKA), receptor protein tyrosine kinase signaling pathway (RTKs), positive regulation of metabolic process (PRM), response to drug (RD), response to stress (RS).

We postulated that the 75 subnetwork genes that were implicated in both data sets and could implicate events that disrupt the protein interaction lattice. In particular, we hypothesized that mutations may have significantly impacted the expression of protein interaction partners and their downstream targets or network affiliations, without affecting the expression of the mutant genes themselves [7, 47]. The majority of the subnetworks we identified consisted of a mixture of genes with and without significantly altered expression levels. For example, although *TP53* displays driver mutations in HGSOC samples[48–50], we did not find significant changes in *TP53* expression between HGSOC and LMP samples. Nevertheless *TP53* was present in subnetworks that distinguished HGSOC from LMP (**Fig 3A**, *P* <10^−4^). Similarly, *BRCA1* [51] participated in the significant differential subnetworks (*P* <10^−4^) but was not differentially expressed (**Fig 3B**). Other examples of driver genes identified in the subnetwork analysis that were not differentially expressed included *ERBB2* [52] (**Fig 3C**), *MYC* (**Fig 3D**) [53–55], and *CTNNB1* (**Fig 3G)** [56] (*P* <10^−4^ for each). Although we did not have the original tumor samples with which to assess the presence of somatic mutations, the disrupted pathway interactions detected by the subnetwork analysis implicate consistent alteration of these gene functions between tumor types. Thus, this network-based method allowed us to assess mutations in the context of networks, enhancing our ability to identify driver mutations.

#### Data interpretation using pathway analysis

To address the biological roles of the subnetwork genes, we performed a GO analysis on all significant subnetworks. A large proportion of GSE9891- and GSE17308-generated subnetworks (59.7% and 52.8%, respectively) were enriched for biological process terms related to cancer, including proliferation, apoptosis, cell cycle, differentiation, kinase activity, stress response, DNA replication, and DNA damage repair. By contrast, only 8.6% and 8.3% of randomly generated networks were associated with these biological processes (P < 2.2*10^−16^, Fisher’s exact test) Additionally, a KEGG pathway analysis showed that 41.5% of the GSE9891 and 28.3% of the GSE17308 significantly altered subnetworks were enriched for *TP53*, *ERBB2*, *MAPK*, B-cell receptor, cell cycle, and focal adhesion pathways, compared with 0.5% and 1% of random networks (P < 2.2*10^−16^, Fisher’s exact test). Importantly, disruption of *TP53*, *ERBB*, and *MAPK* pathways has been implicated in ovarian tumorigenesis [57–60], focal adhesion is the most deregulated pathway in ovarian cancer [61], and B-cell infiltration of ovarian carcinoma effusions is associated with worse outcomes [62]. Thus, the reproducibility of our findings across data sets and their alignment with the existing literature show that an *in silico* approach can be used to implicate subnetworks that play important functional roles in tumorigenesis and identify known processes involved in tumor progression.

### Significantly Modified Hub Protein Interactions

The connectivity of disease-relevant proteins and their interaction partners was further investigated by assessing the involvement of the most highly connected proteins in the cell: the hub proteins. Because these proteins play critical roles in development and reproduction, deleterious mutations are typically lethal [24, 63, 64]. Compared with mutations at the network periphery, loss-of-function mutations in these genes are rare and devastate network functions in cells. However, tumor cells can adapt to a partial loss of hub protein function by rewiring their networks to establish workarounds. To assess altered hub protein function, we applied differential network mapping [65] to our HGSOC and LMP comparison. To do so, we calculated the average Pearson correlation coefficient (PCC) between gene expression values of all hub proteins and their interaction partners, directly comparing LMP and HGSOC samples. We analyzed 3,128 hub proteins that interact with at least 5 other proteins and identified 178 hub proteins in GSE17308 and 220 in GSE9891 whose PCCs differed across tumor types (*P* <0.05); each was connected to genes with measurable expression changes between the tumor types. Of these hub proteins, 34 were implicated in both data sets (**S2 Table**).

**Fig 4:**
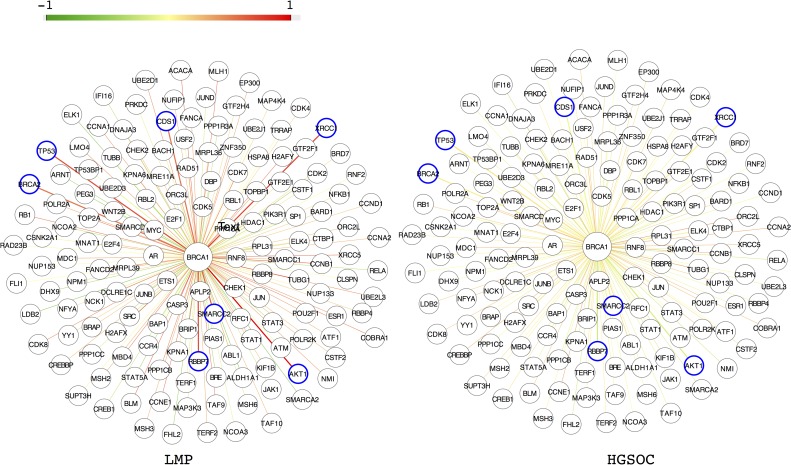
Pearson correlation coefficients (PCCs) for expression levels of *BRCA1* and interaction partners in low-malignant-potential (LMP) and high-grade serous carcinoma (HGSOC) samples. The color of an edge represents the value of the PCC for the genes that it connects. Blue circles indicate genes whose expression levels are highly correlated with *BRCA1* expression levels in LMP, but not in HGSOC.

One of the hub proteins was *BRCA1*. *BRCA1* expression was less correlated with *TP53* and *BRCA2* expression in HGSOC than in LMP samples. The value of the PCC decreased significantly from 0.704 and 0.551 in LMP to 0.096 and 0.065 in HGSOC for *TP53* and *BRCA2*, respectively (P = 0.0023 and P = 0.022, **Fig 4, Table 1**). Several additional genes also had PCCs with *BRCA1* that differed significantly across tumor types, including *AKT1*, which encodes a multifunctional serine-threonine protein kinase; *XRCC1*, a DNA repair gene; and *RBBP7*, *CDS1*, and *SMARCC2*. All of the genes mentioned exhibited decreased expression correlations with *BRCA1* in HGSOC (**Fig 4, Table 1**), suggesting disrupted function of the BRCA1 protein.

**Table 1:** The correlation coefficients of expression levels of *BRCA1* and interaction partners in low-malignant-potential (LMP) and high-grade serous carcinoma (HGSOC) data sets.

	LMP	HGSCO	Z-value*	P-value*
*TP53*	0.704	0.096	2.84	0.0023
*BRCA2*	0.551	0.065	2.02	0.022
*AKT1*	0.577	-0.163	3	0.0013
*XRCC1*	0.746	0.043	3.35	0.0004
*RBBP7*	0.72	0.34	2.02	0.022
*CDS1*	0.55	-0.66	5.14	0
*SMARCC2*	0.623	0.067	2.4	0.008

* Fisher’s z transformation was appled to obtain Z-value and P-value.

Similar to the *BRCA1* results, the vast majority of significant hub proteins in each GEO set were not differentially expressed themselves [205/220 (93.2%) in GSE9891 and 168/178 (94.4%) in GSE17308]. Instead, hub proteins were implicated through differential expression PCCs among interaction partners between tumor types.

#### Data interpretation using pathway analysis

**Fig 5:**
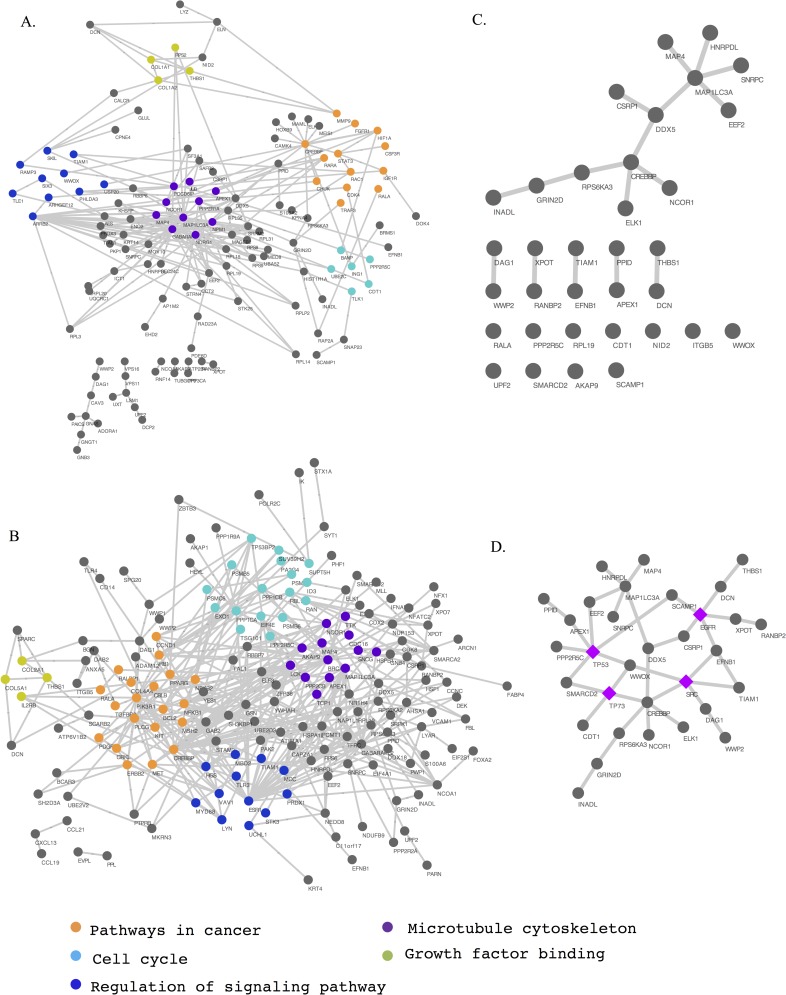
An overarching network connecting hub proteins differentially involved in low-malignant-potential (LMP) and high-grade serous carcinoma (HGSOC) samples. Nodes and edges illustrate the network diagrams of GEO data sets (A) GSE17308 and (B) GSE9891. Each functional complex is represented by a different color. (C) There are 34 hubs that overlap between the two data sets. (D) Additional gene products, highlighted in purple, have the largest number of connections to the 34 concordant hubs, interconnecting all of the hubs except for six orphans.

We traced most of the significant hub proteins detected in GSE17308 (109/178) and GSE9891 (167/220) to an interconnected network enriched for cancer pathways, cell cycle, signaling, and growth factor binding (as defined by IPA: *P* <0.05 for Fisher’s exact test; **Fig 5A and 5B**). Of the 34 hub proteins found in both data sets, 19 are associated with cancer (*P* <0.05), 23 have been linked to genetic disorders (*P* <0.05), and 31 have been associated with the molecular function GO term “protein binding” (*P* <5.3 × 10^−5^). Moreover, 10 of the 34 hub proteins interacted with each other (**Fig 5C**, middle panel; five interacting hub protein pairs), and 13 directly participated in the same interconnected network we derived from known protein interactions, whereas 11 proteins lacked any central ties (**Fig 5C**). Notably, *CREBBP*, whose product plays an essential role in the cell cycle, was frequently interconnected with hubs identified here and also directly interacted with three of the genes differentially expressed in both data sets: *STAT1*, *MYBL2*, and *SOX9*. The published TCGA data have documented five nonsynonymous and two frameshift somatic mutations in *CREBBP* in 316 HGSOC cases [16]. Our data implicate *CREBBP* interactions as a source of differential information trafficking between LMP and HGSOC tumors.

#### Analysis of missing links in network data

To further explore the extent of hub protein disruption, we searched for “bridge” or “broker” proteins [66] that connect isolated hub proteins with mainstream core networks. This analysis revealed that *TP53* connects the largest number of orphan hubs (*n* = 6), with *SRC*, *EGFR*, and *TP73* providing additional connections. The central position of these four proteins in a larger network implicates them in disrupting network lattice interactions (**Fig 5D**). As is true for *TP53*, the documented involvement of these genes in ovarian cancer or tumors in general supports their inclusion as genes of interest [48, 67–69].

### Comparison with Indexed Literature

To explore alternative and complementary approaches to our network analysis, we used the GeneIndexer tool, which reconstructs functional relationships among genes based on extensive literature searches and semantic indexing. We fed our list of 34 hub proteins to GeneIndexer, along with three genes that contain causal mutations in HGSOC genes, either hereditary (*BRCA1* and *BRCA2*) or somatic (*TP53*). Using a functional hierarchy tree built using our list of 34 hub proteins and three disease-associated genes, GeneIndexer indicated that *BRCA1*, *BRCA2*, and *TP53* were the genes from the list with the strongest functional connection to each other (**S5 Fig**). The gene most closely related to these three genes was *APEX1*, a base excision repair gene with elevated or altered expression in breast, cervical, and germ cell tumors; gliomas; rhabdomyosarcomas; and non–small cell lung cancer [70, 71]. *CREBBP*, the fifth most connected gene using the GeneIndexer approach (**S5 Fig**), clustered with *NCOR1* (nuclear receptor corepressor 1) in the functional relationship tree. The close functional relationship between *CREBBP* and *NCOR1* was also demonstrated in our hub protein network (**Fig 5C and 5D**). A number of studies have suggested that *NCOR1* plays an important role in human cancers [16, 72–75].

### Known Genetic Mutations in Concordant Gene Lists

**Fig 6:**
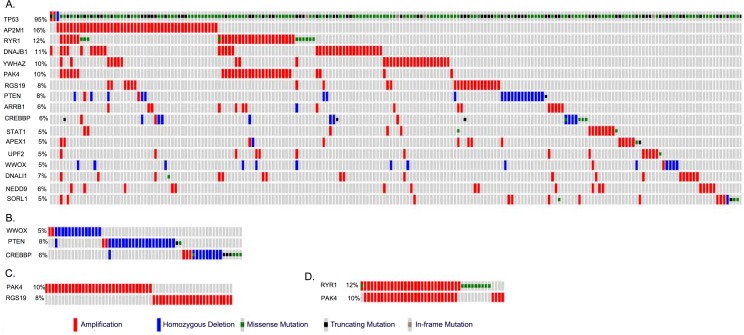
Mutations in genes that significantly differed between low-malignant-potential (LMP) and high-grade serous carcinoma (HGSOC) samples in two data sets, GSE17308 (13) and GSE9891 (16), using gene expression, network, or hub protein analyses. (A) At least 5% (16/316) of Cancer Genome Atlas HGSOC samples contained one or more mutant versions of 17 genes identified from differential expression, network, or hub protein analyses. (B) The majority of genetic alterations in *WWOX*, *PTEN*, and *CREBBP* in HGSOC samples were homozygous deletions. (C) Amplifications in *PAK4* and *RGS19* tended to be mutually exclusive, although this association was not statistically significant (*P* <0.07). (D) Amplifications in *PAK4* and *RYR1* frequently co-occurred (*P* <0.001).

Using TCGA data, which contain information on somatic mutations and copy number variation for HGSOC patient samples, we searched for genetic mutations occurring in our concordant differentially expressed genes, significant subnetwork genes, and genes encoding significant hub proteins. The somatic mutations were identified using a combination of three algorithms, VarScan 2, SomaticSniper and GATK, applied to 316 high-grade serous carcinomas (HGSOC) samples and 236 normal tissue samples in the TCGA project[16]. Variants were annotated as somatic mutations if they were not observed in the normal samples [16]. We found that 17 of those genes were somatically mutated in HGSOC cases, and that each of these mutant genes was present in 5 to 95% (or 16 to 300 of 316) of patient samples (**Fig 6A**). For example, *TP53* somatic mutations were found in 95% (300/316) of HGSOC samples. When *TP53* was removed from consideration, 67% (210/316) of the samples contained one or more somatic mutations from the 16 remaining concordant genes. We also assessed the presence of amplification and deletion events among the genes implicated in our analyses. Collectively, homozygous deletions occurred in *PTEN*, *CREBBP*, and *WWOX* in 43/316 (13.7%) of HGSOC samples, whereas amplifications of *AP2M1*, *RYR1*, *DNAJB1*, *YWHAZ*, *PAK4*, *RGS19*, *ARRB1*, *STAT1*, *APEX1*, *UPF2*, *DNALI1*, *NEDD9*, and *SORL1* were found in 179/316 (56.7%) of HGSOC samples (**Fig 6A and 6B**). Notably, *RYR1* and *APEX1* are the targets of two FDA-approved cancer drugs (caffeine and lucanthone, respectively). We also found that amplification of *PAK4* and *RGS19* tended to be mutually exclusive, although this trend did not reach statistical significance, (*P* = 0.07, Fisher’s exact test, **Fig 6C**), whereas amplification of *RYR1* and *PAK4* tended to co-occur, an association that did reach statistical significance (*P* <0.001, Fisher’s exact test, **Fig 6D**).

**Fig 7:**
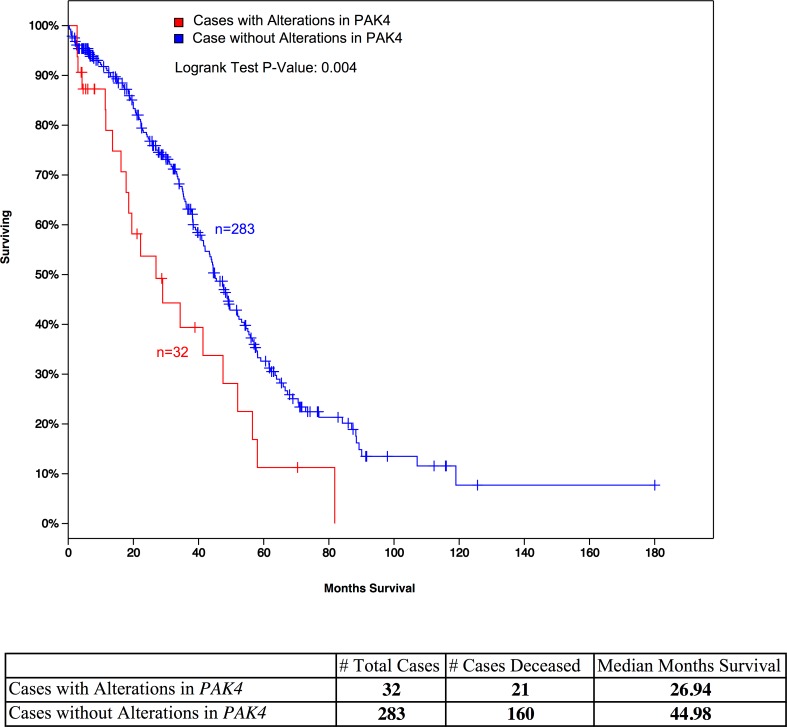
A Kaplan-Meier survival analysis of HGSOC patients with *PAK4* amplification. HGSOC patients (*n* = 32) with *PAK4* amplification have a significantly shorter survival time (*P* = 0.004) than patients without *PAK4* amplification (*n* = 283).

The known roles of these proteins in tumorigenesis support our predictions of their relevance. For example, *PAK4* mutations promote oncogenic transformation, and *PAK4* deletions inhibit tumorigenesis [76, 77], whereas *RGS19* deregulates cell proliferation through multiple pathways [78]. In addition, we performed a univariate survival analysis for 17 significant genes identified by our study and also displayed somatic mutations reported by TCGA project in more than 5% HGSOC patients. The *PAK4* has lowest P-value in the survival analysis (P = 0.004, P-adjust = 0.068) (**Fig 7**). Despite amplification of *RYR1* and *PAK4* tended to co-occur, survival times in patients with *RYR1* alterations alone were not significantly shorter (P = 0.18). Both *RYR1* and *PAK4* participate in diverse functional pathways in the cell. *RYR1* is present in several KEGG pathways, including calcium signaling, circadian entrainment, long-term depression, and oxytocin signaling. *PAK4* is present in pathways for renal cell carcinoma, ErbB signaling (**S6 Fig**), focal adhesion, T-cell receptor signaling, and regulation of the actin cytoskeleton. Although *RYR1* and *PAK4* are not known to act in the same pathways, their observed co-occurrence in our PPI data suggests that these two genes could interact indirectly through other proteins (**S7 Fig**).

### Comparison of Differentially Expressed Genes, Subnetworks, and Significant Hubs

To evaluate the effectiveness of the various approaches to identifying disease genes used in this study, we compiled a list of 18 known ovarian cancer susceptibility genes present in the GEO expression data sets; we identified these genes by searching for known somatic and germline mutations in ovarian cancer from the Online Mendelian Inheritance in Man (OMIM) database. We identified 195 and 230 differentially expressed genes, 179 and 175 differential subnetworks that consist of 502 and 397 genes, and 178 and 220 significant hubs, for GSE17308 and GSE9891, respectively (**S8 Fig**). The comparison of our findings with this known list showed that the subnetwork analysis identified nine of these known genes (50%), the hub protein analysis identified five (27.8%), and the differential expression analysis identified one (5.6%). The difference in sensitivity between these methods was not statistically significant (*P* = 0.18 for the subnetwork vs. differentially expressed genes comparison, *P* = 0.1 for the hub vs. differentially expressed genes comparison, Fisher exact test). However, *TP53*, *BRCA1*, *ERBB2*, *MLH1*, *PIK3CA*, and *RAD51C* were detectable only with the two network-based approaches. When we examined the methods’ ability to detect a list of 288 genes from the Catalogue of Somatic Mutations in Cancer (COSMIC) database that display somatic and germline mutations in general cancer, our subnetwork (21.1%; *P* = 0.01, Fisher exact test) and hub approaches (12.5%, *P* = 0.07) identified significantly or nearly significantly more genes than did the differential expression analysis (5.2%; **S9 Fig**). This result indicates that network-based approaches may be more useful in terms of identifying important cancer-associated genes and aiding in hypothesis generation than differential expression analyses.

### Blind Classification Using a Support Vector Machine Classifier

Finally we constructed support vector machine (SVM) classifiers, which adopted different types molecular signatures (i.e., subnetworks, hub proteins, and differential gene expression, details in the Materials and Methods section) as input features, to assess and compare their effectiveness in classifying blinded sets of HGSOC and LMP tumor samples. Performance was evaluated using threefold cross-validation with bootstrap sampling. Regardless of the molecular signature used in the classification, we achieved robust separation of the samples, as shown by area under the ROC curve (AUC) values **(S10 Fig, S3 Table).** AUCs reached 0.98 for both intra-data set classification, in which the molecular signatures used for training and classification were derived from the same data set, and inter-data set classification, in which the molecular signatures used for training and classification were derived from different data sets. The successful classification of HGSOC and LMP samples suggests that these signatures capture the molecular perturbations and alterations occurring in the tumors and, moreover, that the pathways that underlie the initiation and progression of LMP and HGSOC are different.

## Discussion

We have extended traditional gene expression analyses by incorporating a systems biology approach to dissect the molecular differences between LMP and HGSOC. Although the differentially expressed genes that we identified characterized distinct properties of the tumors, the cause-and-effect relationships underlying transcriptional disruption could not be explained with gene expression data alone. Our subnetwork analysis identified a series of significantly altered, interacting complexes that coordinate higher-level functions in important biological pathways, demonstrating that differential gene expression reflects multiple processes in tumor cells. In addition, our hub protein analysis identified consistent alterations in the information-dissemination centers that perpetuated the largest numbers of downstream events. Many hub proteins involve in multiple signaling pathways such as P53 and BRCA1. Mutation of the genes encoding these hub proteins could affect multiple pathways. Thus, targeting these genes could simultaneously activate or inhibit disease pathways.

In this analysis, 6 of 23 differentially expressed between LMP and HGSOC samples in two independent patient data sets also participated in subnetworks that were altered between tumor types in both data sets. Hence, these six genes (*STAT1*, *MYBL2*, *RPS23*, *NR2F1*, *SOX9*, and *SPRY2*) merit the highest priority for further study. Furthermore, our results suggest several hypotheses regarding disease mechanisms. For instance, *CREBBP* (*CBP*) interactions with *STAT1* and *MYBL2* appeared in three altered subnetworks, suggesting that each of these subnetworks is disrupted through *CREBBP* dysfunction. *CREBBP* mutations are documented in HGSOC (11), and our results suggest that *CREBBP* may contains causative mutations or homozygous deletions in the expression data sets we studied.

On the basis of a static GO analysis of differentially expressed genes concordant in both GEO data sets, we infer that RAR activation and AhR signaling pathways may be disrupted in HGSOC. However, our dynamic subnetwork analysis suggests that other pathways are differentially affected as well, including the P53, ERBB, chemokine, MAPK, and B-cell receptor signaling pathways. Consistent with our results, the P53, ERBB, and MAPK signaling pathways have documented regulatory roles in ovarian carcinomas (63–66). In addition, several genes mutated in HGSOC, such as *TP53*, *BRCA1*, and *MYC*, were identified as participants in significant subnetworks. Our results suggested that the network method, which combines expression profile and protein-protein interactions, can infer disease genes carried causative mutations and regulated the expression levels of the downstream genes.

In addition, by combining our network analysis results with HGSOC genotype data from TCGA, we found co-occurring mutations in *RYR1* (which encodes the ryanodine receptor) and *PAK4*, a result with potential clinical applications. *RYR1* alteration has been implicated in various types of disease, including cancer [79]. Presently, *RYR1* is a target of four FDA-approved drugs, including procaine, dantrolene, suramin, and caffeine (**S7 Fig**). Our survival analysis suggests that HGSOC patients with *PAK4* mutations have poor survival rates, but currently there is no drug that specifically targets *PAK4*, and it is often very expensive and time-consuming to develop a new therapeutic. As mutations of *PAK4* and *RYR1* tend to co-occur in HGSOC, and the two genes might interact, we hypothesize that caffeine, a cancer drug that targets *RYR1* [80] (**S7 Fig),** may represent a useful intervention strategy to treat HGSOC patients with *PAK4* mutations. Caffeine has been reported to impact cell cycle function, trigger apoptosis or programmed cell death or and disturbe key cell cycle regulatory proteins[81]. In addition, *PAK4* lies downstream of EGFR in the ERBB signaling pathway; hence, the nine FDA-approved cancer drugs targeting EGFR should be assessed to see if they ameliorate the effects of *PAK4* mutations and could be used to treat HGSOC patients.

A previous study reported that LMP affects women at a younger age than invasive ovarian cancer [82]. Consistently, the age distribution of patients of the two datasets (GSE17308 and GSE9891) showed a similar trend. For GSE17308, LMP patients ranged in age from 25 to 76 years (mean = 52 years), whereas HGSOC patients ranged in age from 25 to 80 years (mean = 62 years). For GSE9891, LMP patients ranged in age from 22 to 79 years (median = 50 years), whereas HGSOC patients ranged in age from 23 to 80 years (median = 59 years). In the original study that analyzed GSE9891[12], the author found that the age difference between the subtypes was significantly different (Kruskal-Wallis test for age as continuous variable, *P* = 0.003). However, the original study that analyzed GSE17308 showed that the difference did not quite reach significance (*P* = 0.08, Kruskal-Wallist test) and had no observable effect on gene expression profiles in unsupervised clustering analysis or in supervised ANOVA analyses [9]. The age difference might impact the gene expression analysis in some datasets. However, in this study our goal is to identify the common differentially expressed genes. We found that the common differentially expressed genes of the two datasets (GSE17308 and GSE9891) successfully clustered LMP and HGSOC patients in the third independent data (GSE27651, **S3 Fig**), suggesting the age influence on gene expression analysis is minimized in our approach.

In this study, network methods implicated a larger number of known ovarian cancer susceptibility genes than differential expression analysis. Given that ovarian cancer is a highly heterogeneous disease, the insights gained through network analyses may improve our understanding of the biological mechanisms involved in disease development and progression. Comparisons between LMP and HGSOC tumors using network approaches indicate that cellular regulatory pathways are wired differently between these tumor types. By considering LMP samples as the reference group, our results provide insight into the mechanisms responsible for the formation and progression of malignant ovarian cancer. More broadly, our work demonstrates that differential gene expression translates into altered network communication at the protein level. Hence, network models, which integrate multilayer information, foster the identification of genomic mutations and aberrant pathways, while facilitating the development of strategies for disease detection and points of intervention.

### Conclusions

A comprehensive catalog of biomarkers is critical for improving our understanding and treatment of HGSOC. Our systematic, systems biology comparison of LMP and HGSOC tumors provides new insights into probable mechanisms underlying malignancy. Integrating gene expression profiles from two independent patient data sets with PPI data allowed us to identify a set of biomarkers that can be used to distinguish between the two tumor types. We then applied these molecular signatures to a third, independent data set to demonstrate their utility. In addition, by combining information on networks altered between the two types of tumors with mutation data, we were able to prioritize mutations for further examination. Our results underscore the strength of systems biology approaches in implicating novel disease mechanisms, and our network approach is especially valuable when a single type of tumor displays recurrent alterations to the same pathways but varies in terms of the individual mutations responsible.

## Materials and Methods

### Data Availability and Online Tools

Subnetwork data have been deposited on the website http://mqyang.net/CancerResearch/HGSOC_Biomarker2.cgi. A web tool that allows users to stratify ovarian cancer samples on the basis of expression data, are available on our website http://mqyang.net/CancerResearch/ClusterTissues.cgi. Supplemental data including our gene lists and related tools are available at http://mqyang.net/CancerResearch/HGSOC_Biomarker2.cgi.

Disease-related subnetwork and hub proteins are searchable on the website, and data from external databases, such as OMIM and TCGA, are linked to the query genes as well. In addition, each gene is linked to external databases, including the UCSC genome browser, KEGG pathways, and the genetic mutations identified in TCGA.

### Expression profiles

Two ovarian tumor expression profiles, GSE17308 [9] and GSE9891 [12], were obtained from the GEO database. In GSE17308, microarray expression profiling was conducted using the PC human Operon 21k v2 platform; 7 LMP and 22 HGSOC samples were collected from patients who were diagnosed with ovarian cancer and treated at the Royal Brisbane and Women's Hospital. LMP patients ranged in age from 25 to 76 years (mean = 52 years), whereas HGSOC patients ranged in age from 25 to 80 years for GSE17308. Experienced pathologists independently reviewed all tumor tissues. GSE9891 contains 18 LMP and 118 HGSOC samples from the AOCS (Australian Ovarian Cancer Study); here, profiling was carried out on well-characterized ovarian cancer tissues from patients with the Affymetrix U133_plus2.0 platform. LMP patients ranged in age from 22 to 79 years (median = 50 years), whereas HGSOC patients ranged in age from 23 to 80 years (median = 59 years) for GSE9891. Because two distinct array platforms were compared, official gene symbols were used to identify genes present in both data sets. Duplicate genes and low-quality data were removed from the analysis, leaving a total of 9,016 genes present in both profiles for evaluation. A third expression profile, GSE27651 [29], was used for validation. It contained 8 LMP and 24 HGSOC samples from the archives of the Department of Pathology at The University of Texas MD Anderson Cancer Center (Houston, Texas). This profile was generated with the commercial GeneChip Human Genome U133 Plus 2.0 Array [29].

### Reproducibility of Differentially Expressed Genes

Four methods of ranking differentially expressed genes in the GSE17308 and GSE9891 data sets were compared: (i) fold change calculated with sample means, (ii) fold change calculated with sample medians, (iii) t-test *P*-values, and (iv) Wilcoxon rank-sum test *P*-values. After genes were ranked either by *P*-value or fold change, the reproducibility rate was calculated as the percent overlap of top-ranked genes in the independent GEO profiles. To estimate the null reproducibility rate, we randomly extracted genes from both profiles for the overlap calculation 1,000 times.

### Human Protein-Protein Interaction Data

We combined human PPI data from five public databases: IntAct, MINT, BioGrid, DIP, and HPRD [82–86]. Each database contains PPIs curated by experts. After removing redundant entries, we obtained a total of 122,403 unique human PPIs.

### Construction of Distinguishing Subnetworks

To construct a subnetwork, expression levels of genes in the profile were first normalized so that the mean and variance across samples were 0 and 1, respectively:

$${\text{Z}}_{\text{i},\text{j}}=\frac{{\text{x}}_{\text{i},\text{j}}\text{-}\mu_{\text{i}}}{\sigma_{\text{i}}}$$ (1)

where *Z*_*i*,*j*_ represents the normalized expression value of the *ith* gene for the *jth* sample. The expression level *Z*_*j*_ of a network of *n* interacting genes was obtained by averaging expression values over all *n* genes in the *jth* sample, as follows:

$${\bar{\text{Z}}}_{\text{j}}=\frac{1}{\text{n-}1}\sum_{\text{i}=1}^{1}{\text{Z}}_{\text{i},\text{j}}$$ (2)

We then used mutual information to estimate the ability of each subnetwork to identify distinct phenotypes. Mutual information quantifies the degree to which two random variables are independent. When a random variable, *X*, is independent of another random variable, *Y*, *I (X; Y)* = 0. When applied to subnetworks, the larger the mutual information value, *MI*, the greater the discrimination power of the subnetwork. Thus, the DS of a subnetwork was defined as *MI*, given by

$$\text{MI}(\text{X};\text{Y})=\sum_{\text{x}\in \text{X}}\sum_{\text{y}\in \text{Y}}\text{P}\{\text{X}=\text{x},\;\text{Y}=\text{y}\}{\text{log}}_{2}\left(\frac{\text{P}\{\text{X}=\text{x},\text{Y}=\text{y}\}}{\text{P}\{\text{X}=\text{x}\}\text{P}\{\text{Y}=\text{y}\}}\right)$$ (3)

where *X* refers to the average normalized expression level of the subnetwork and *Y* refers to the tissue phenotype. In the equation above, *X* is assumed to take discrete values, but the average normalized expression level, *Z*, defined by (2), is not a discrete variable. We therefore discretized *Z* by dividing its range into equally spaced bins defined by split points, *s*_*k*_, resulting in the following expression for the DS:

$$\text{DS}(\bar{\text{Z}};\text{Y})=\sum_{\text{k}=1}^{\text{m}}\sum_{\text{y}\in \text{Y}}\text{P}\{{\text{s}}_{\text{k}}<\bar{\text{Z}}\leq {\text{s}}_{\text{k}=1},\text{Y}=\text{y}\}{\text{log}}_{2}(\frac{\text{P}\{{\text{s}}_{\text{k}}<\bar{\text{Z}}\leq {\text{s}}_{\text{k}=1},\text{Y}=\text{y}\}}{\text{P}\{{\text{s}}_{\text{k}}<\bar{\text{Z}}\leq {\text{s}}_{\text{k}+1}\}\text{P}\{\text{Y}=\text{y}\}})$$ (4)

To cover all values, we took *s*_*1*_ *= min(Z) − δ*, and *s*_*m+1*_ *= max(Z) + δ*. The number of bins was *m =* log_2_(# samples) + 1.

The growth of each subnetwork was guided by a greedy algorithm in an iterative procedure. At each iteration, genes that neighbored at least one gene in the network were candidates for addition.

For each candidate gene, the DS was evaluated using the average expression of that gene and the genes in the current subnetwork. Among all candidate genes, the one that generated the largest DS was selected and added to the current network. The search procedure was terminated when the improvement rate, defined as the ratio of the winning DS for successive iterations, either increased or was <0.1, or if the distance between the winning gene and the seed of the network was >2.

To test the hypothesis that a subnetwork could distinguish between different phenotypes, we obtained two DS null distributions by selecting two groups of genes with the same number of members as the given subnetwork, one with the same seed protein and the other with a different seed protein; we repeated this process 10,000 times. To test the hypothesis that genes in a subnetwork were associated with a particular phenotype, we constructed a DS null distribution by randomly permuting the phenotypes of tissues 10,000 times. Subnetworks with *P*-values <10^−4^ in both tests and DS >0.66 were selected.

### Identification of Significant Hub Proteins

Hub proteins were required to have at least five interactions. These hubs represented approximately the top 20% of all proteins in terms of number of interactions. For each hub (*H*), the difference in the PCC between LMP and HGSOC samples for an individual interaction (*I*) was calculated as follows:

$$\nabla {\text{r}}_{\text{H},\text{I}}=\frac{\sum_{\text{j}}\left({\text{I}}_{\text{j},\text{L}}\text{-}{\bar{\text{I}}}_{\text{L}})({\text{H}}_{\text{j},\text{L}}\text{-}{\bar{\text{H}}}_{\text{L}}\right)}{({\text{n}}_{\text{L}}\text{-}1){\text{S}}_{{\text{I}}_{\text{L}}}{\text{S}}_{{\text{H}}_{\text{L}}}}\text{-}\frac{\sum_{\text{j}}\left({\text{I}}_{\text{j},\text{C}}\text{-}{\bar{\text{I}}}_{\text{C}})({\text{H}}_{\text{j},\text{C}}\text{-}{\bar{\text{H}}}_{\text{C}}\right)}{({\text{n}}_{\text{C}}\text{-}1){\text{S}}_{{\text{I}}_{\text{C}}}{\text{S}}_{{\text{H}}_{\text{C}}}}$$ (5)

,where *L* and *C* denote LMP and HGSOC, respectively, and *S* represents the standard deviation. The average of these PCC differences is given by

$$\text{AvgPCC}=\frac{1}{\text{m-}1}\sum_{\text{i}=1}^{\text{m}}\left|\nabla {\text{r}}_{\text{H},{\text{I}}_{\text{i}}}\right|$$ (6)

where *m* is the total number of interactions of the hub. To test the hypothesis that hub protein modularity is significantly altered by disease type, we constructed a null distribution by randomly shuffling the tissue phenotypes 1,000 times. Hubs with *P*-values <0.05 were selected for our study. In addition, by searching known PPIs, the proteins that had the most frequent interactions with isolated hub proteins or protein clusters were identified and used to bridge these isolated instances into a larger network.

### Cross-check with Literature, Pathway Databases and Know Disease Genes

We employed the GeneIndexer webtool (http://geneindexer.com/) to examine and validate functional relationships among the significant hub proteins implicated in both data sets, as well as three well-known cancer genes (*TP53*, *BRCA1 and BRCA2*). On the basis of the scientific literature, GeneIndexer generates a tree by clustering functionally related genes together. In addition, we performed a hypergeometric test to identify the KEGG pathways enriched in gene clusters from the subnetwork analysis. The hypergeometric test was also used to find GO terms that were significantly associated with each discriminative subnetwork. Canonical pathways enriched by differentially expressed genes were detected using Ingenuity’s IPA software.

We identified our list of 18 known ovarian cancer susceptibility genes by downloading gene map file from the OMIM database and searching on the phrase “ovarian cancer susceptibility”. The 288 cancer genes were obtained directly from the COSMIC database.

### Genetic Mutations in Differentially Expressed Genes and Significant Hub Proteins

Genetic mutations in differentially expressed genes and significant hub proteins, which were concordant between expression data sets, were detected using mutation data from a total of 316 sequenced HGSOC tissue samples from TCGA [16]. The statistical significance of the mutual exclusivity and co-occurrence of genetic mutations in gene pairs was assessed using Fisher’s exact test. We used the cBioPortal [87, 88] tool to perform these analyses.

### Prediction of Tumor Types

To evaluate the prediction performance of the molecular signatures identified, we randomly selected two-thirds of the data for training and used the remaining one-third for testing; we repeated this procedure 50 times. The ROC curve and the AUC were plotted and analyzed using the R package ROCR [89]. The features used for classification for the three different types of molecular signatures were calculated as followings: (1) the expression levels of differentially expressed genes, (2) the average expression level of genes comprising discriminative subnetworks, (3) the expression correlation changes of significant hub proteins with their interacting genes.

### Statistical Tests and Network Visualization

We used the R software package to perform statistical tests. Cytoscape 2.8.1 [90] was used to visualize networks.

## Supporting Information

S1 Fig Low-malignant-potential (LMP) and high-grade serous carcinoma (HGSOC) samples, clustered based on the expression levels of top-ranked, differentially expressed genes identified using Wilcox rank-sum test *P*-values or median fold-change. (A) represents tissue clusters for expression data sets GSE9891 (*top*) and GSE17308 (*bottom*) obtained using expression levels of top genes ranked by the Wilcox rank-sum test. (B) represents tissue clusters for GSE9891 (*top*) and GSE17308 (*bottom*) obtained using expression levels of top genes ranked by median fold-change.
(PDF)

S2 Fig The *P*-values and median fold changes for gene expression in low-malignant-potential (LMP) and high-grade serous carcinoma (HGSOC) samples in the GEO data sets GSE17308 (*left*) and GSE9891 (*right*). The red circle represent the top differentially expressed genes selected by P-value and fold change.
(PDF)

S3 Fig Hierarchical cluster analysis on an independent group of samples. Based on expression levels of 23 genes differentially expressed in low-malignant-potentihujujiijnal (LMP) and high-grade serous carcinoma (HGSOC) samples in both the GSE9891 and GSE17308 data sets, samples from a third, independent patient data set (GSE27651) were separated into two homogenous sets. (A) represents a heatmap of tissue clusters for the GSE27651 data set, and (B) represents a hierarchical tree of the same data.
(PDF)

S4 Fig The cell cycle, drug metabolism, and molecular transport network is enriched with genes differentially expressed between low-malignant-potential (LMP) and high-grade serous carcinoma (HGSOC) samples in both the GSE9891 and GSE17308 data sets. Red indicates overexpression in HGSOC and green represents underexpression. Genes highlighted in yellow participate in RAR activation and AhR signaling.
(PDF)

S5 Fig The functional relationships among genes that encode common protein hubs differentially expressed between low-malignant-potential (LMP) and high-grade serous carcinoma (HGSOC) samples in both the GSE9891 and GSE17308 data sets. Using GeneIndexer, which can search over one million Entrez Gene abstracts to identify mechanistic functional relationships among genes, a functional hierarchy tree was constructed. It suggests that the known ovarian cancer genes *BRCA1*, *BRCA2*, and *TP53* have the strongest functional relationships with each other, followed by *APEX1*, a protein hub gene identified in this study.
(PDF)

S6 Fig ERBB signal pathway. *PAK4* is located downstream of *EGFR* in the ERBB signalling pathway.
(PDF)

S7 Fig *PAK4* and *RYR1* protein interaction networks suggest that the two proteins interact indirectly. The yellow octagons respresent FDA approved drugs target the corresponding gene.
(PDF)

S8 Fig The total number of molecular signatures by differential methods. (PDF)

S9 Fig Comparison of the ability of differentially expressed genes, subnetwork connections, and significant hubs to identify known cancer genes. We compiled a list of ovarian cancer susceptibility genes affected by somatic and germline mutations from the OMIM database, and also a list of general cancer genes that carry somatic and germline mutations from the COSMIC database. Subnetwork and hub protein analyses did not reveal significantly more known cancer genes than differential expression analysis in the ovarian cancer gene set, but they did reveal significantly more of the general cancer genes.
(PDF)

S10 Fig ROC curves of support vector machine (SVM)-based classifiers. The purple line represents the classification performance ROC curve for expression profile data set GSE9891, whereas the green line represents that for expression profile data set GSE17308.
(PDF)

S1 Table The significant subnetworks that differentiate HGSOC from LMP. (DOCX)

S2 Table The common significant hub proteins. (DOCX)

S3 Table The performance of classification using different type molecular signature as features. (DOCX)

## Abbreviations

GEO: Gene Expression Omnibus
HGSOC: high-grade serous ovarian carcinoma
LMP: low-malignant-potential tumors
PPI: protein-protein interaction
TCGA: The Cancer Genome Atlas.
PCC: Pearson Correlation Coefficient
AUC: area under curve
GO: Gene Ontology
OMIM: Online Mendelian Inheritance in Man
COSMIC: Catalogue of Somatic Mutations in Cancer
SVM: Support Vector Machne
