# Supplementary figures and images for: A Systems Biology Comparison of Ovarian Cancers Implicates Putative Somatic Driver Mutations through Protein-Protein Interaction Models
Source: PLoS One. 2016 Oct 27;11(10):e0163353. doi: 10.1371/journal.pone.0163353 (PMC5082879; doi:10.1371/journal.pone.0163353)

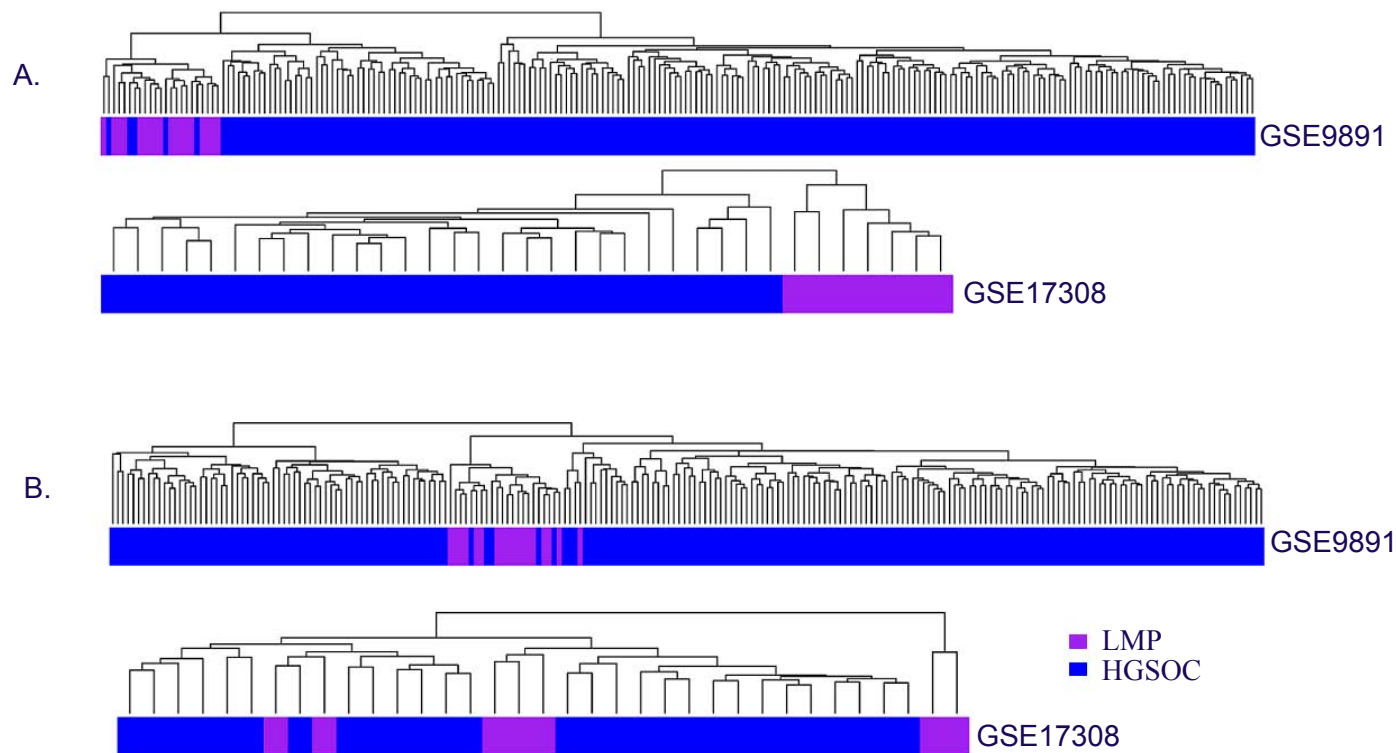

S1 Fig

Supplement: S1 Fig — (A) represents tissue clusters for expression data sets GSE9891 (top) and GSE17308 (bottom) obtained using expression levels of top genes ranked by the Wilcox rank-sum test. (B) represents tissue clusters for GSE9891 (top) and GSE17308 (bottom) obtained using expression levels of top genes ranked by median fold-change. (PDF) [file pone.0163353.s001.pdf]

**GSE17308**

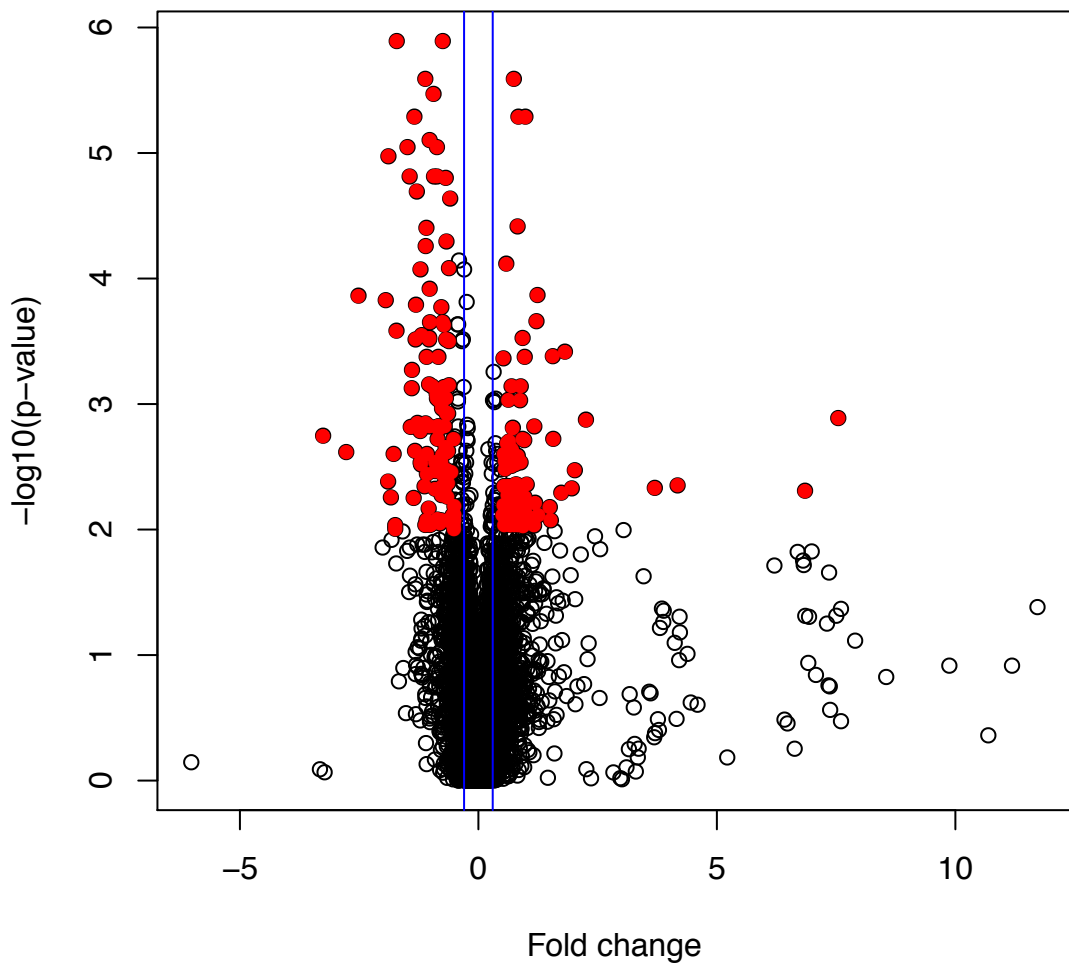

**GSE9891**

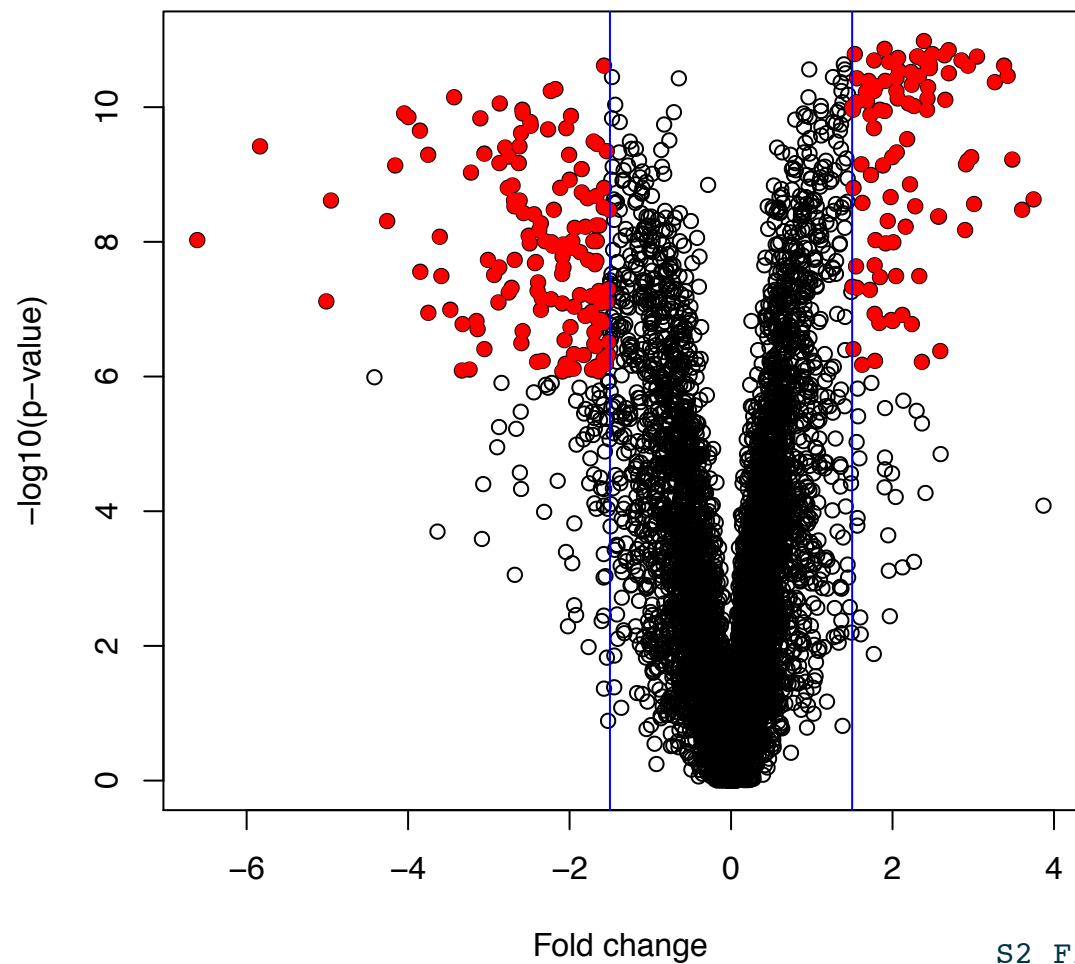

Supplement: S2 Fig — The red circle represent the top differentially expressed genes selected by P-value and fold change. (PDF) [file pone.0163353.s002.pdf]

# GSE27651\_Cluster\_ByCommon\_Gene

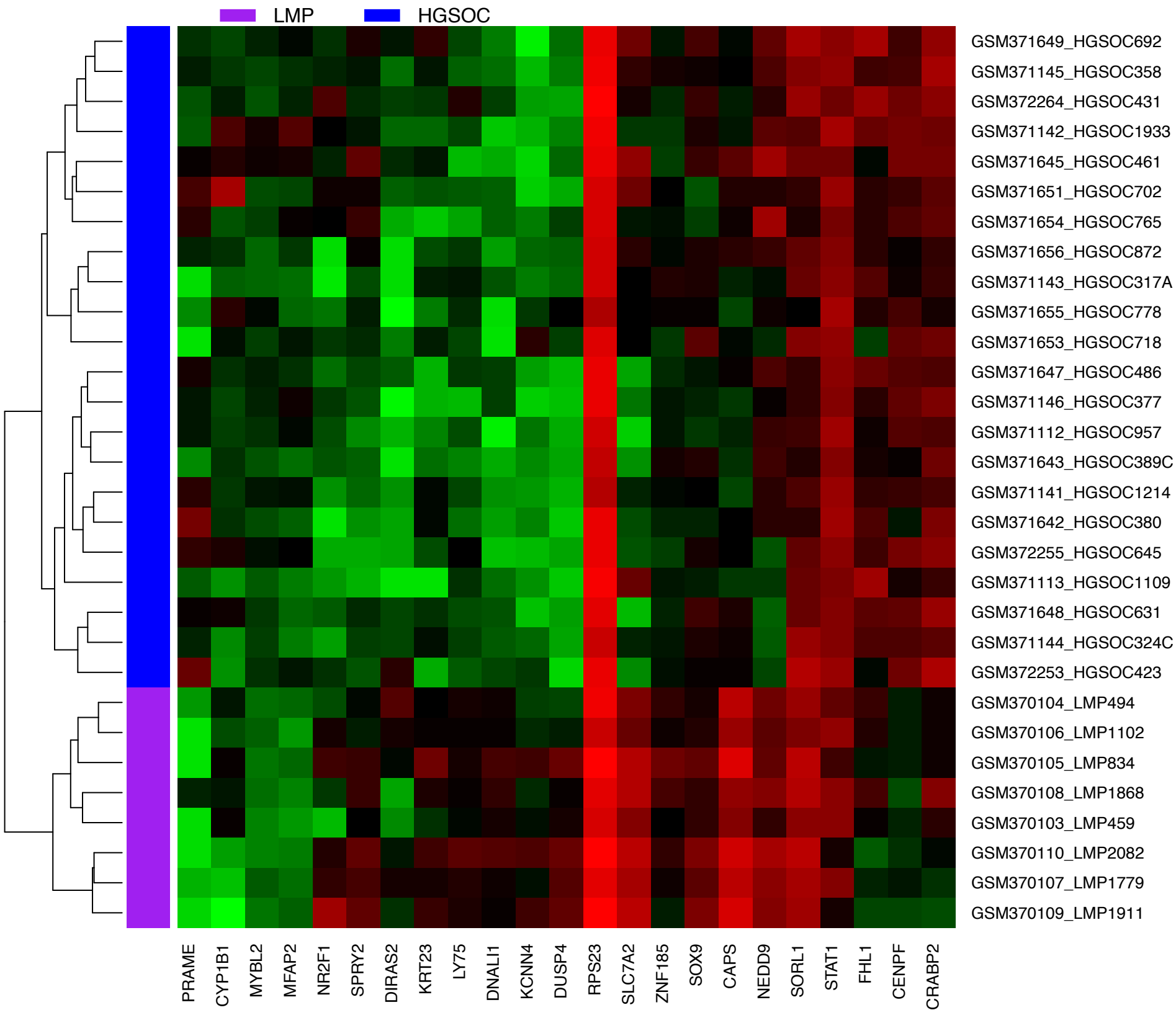

Supplement: S3 Fig — Based on expression levels of 23 genes differentially expressed in low-malignant-potentihujujiijnal (LMP) and high-grade serous carcinoma (HGSOC) samples in both the GSE9891 and GSE17308 data sets, samples from a third, independent patient data set (GSE27651) were separated into two homogenous sets. (A) represents a heatmap of tissue clusters for the GSE27651 data set, and (B) represents a hierarchical tree of the same data. (PDF) [file pone.0163353.s003.pdf]

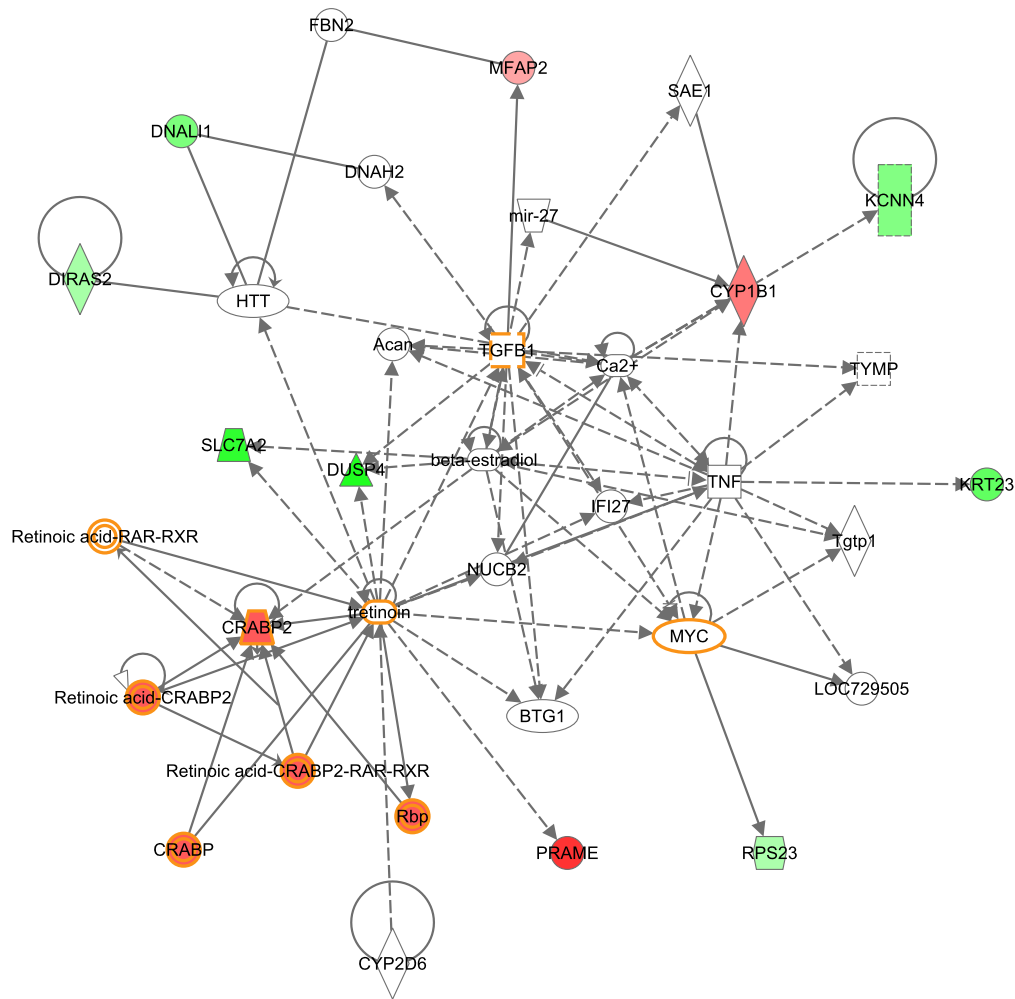

S4 Fig

Supplement: S4 Fig — Red indicates overexpression in HGSOC and green represents underexpression. Genes highlighted in yellow participate in RAR activation and AhR signaling. (PDF) [file pone.0163353.s004.pdf]

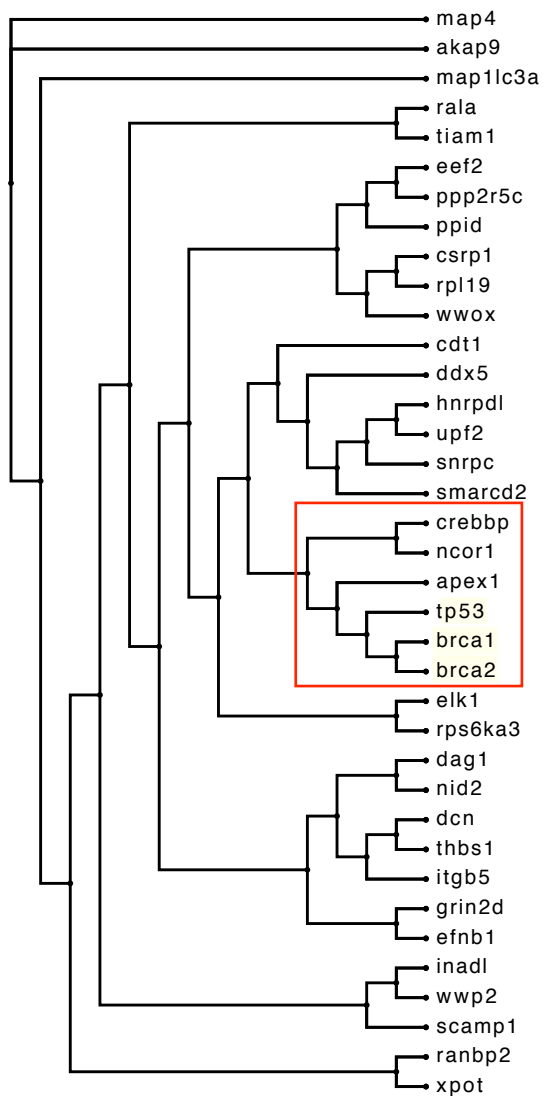

S5 Fig

Supplement: S5 Fig — Using GeneIndexer, which can search over one million Entrez Gene abstracts to identify mechanistic functional relationships among genes, a functional hierarchy tree was constructed. It suggests that the known ovarian cancer genes BRCA1, BRCA2, and TP53 have the strongest functional relationships with each other, followed by APEX1, a protein hub gene identified in this study. (PDF) [file pone.0163353.s005.pdf]

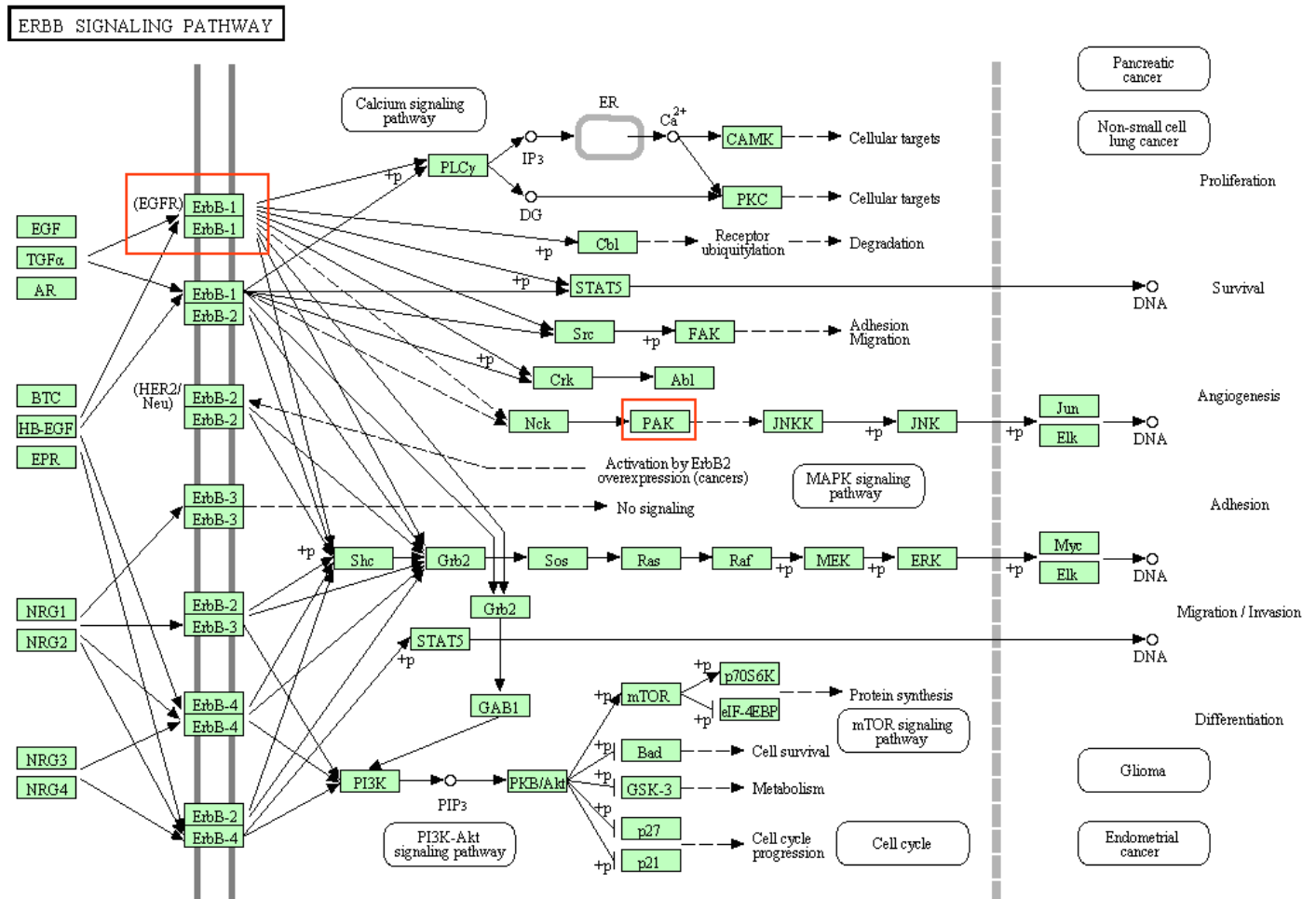

S6 Fig

Supplement: S6 Fig — PAK4 is located downstream of EGFR in the ERBB signalling pathway. (PDF) [file pone.0163353.s006.pdf]

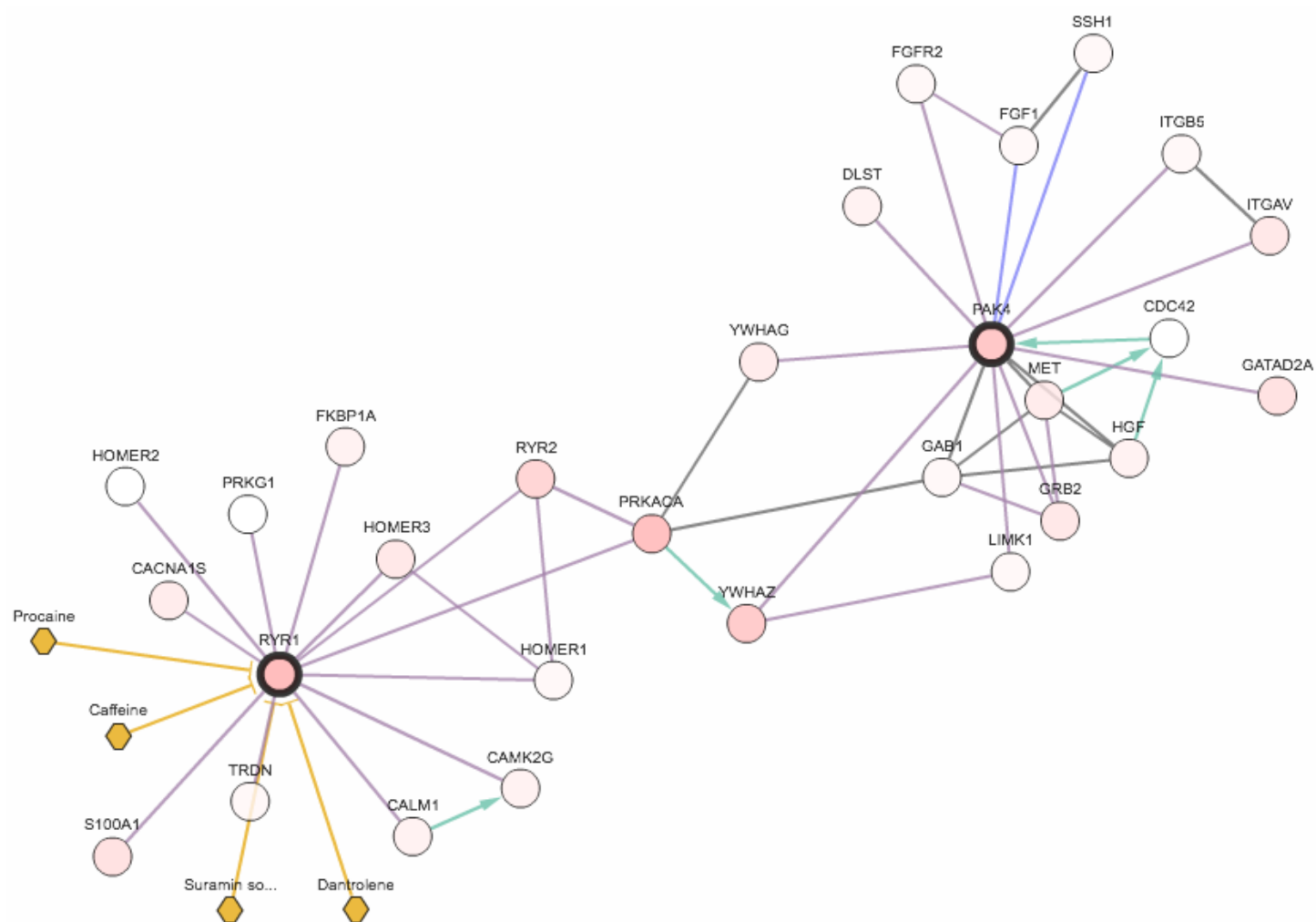

S7 Fig

Supplement: S7 Fig — The yellow octagons respresent FDA approved drugs target the corresponding gene. (PDF) [file pone.0163353.s007.pdf]

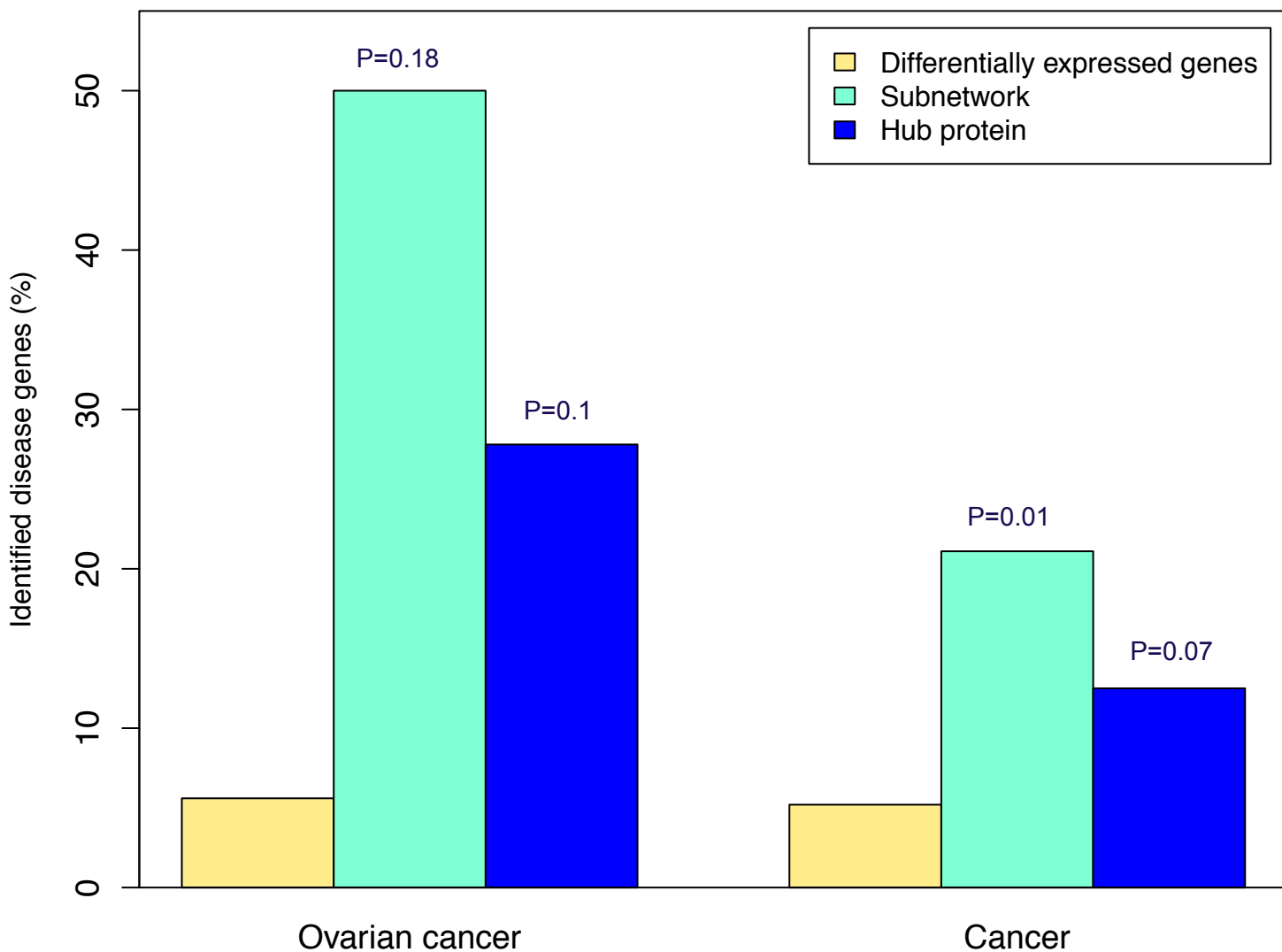

Supplement: S9 Fig — We compiled a list of ovarian cancer susceptibility genes affected by somatic and germline mutations from the OMIM database, and also a list of general cancer genes that carry somatic and germline mutations from the COSMIC database. Subnetwork and hub protein analyses did not reveal significantly more known cancer genes than differential expression analysis in the ovarian cancer gene set, but they did reveal significantly more of the general cancer genes. (PDF) [file pone.0163353.s009.pdf]

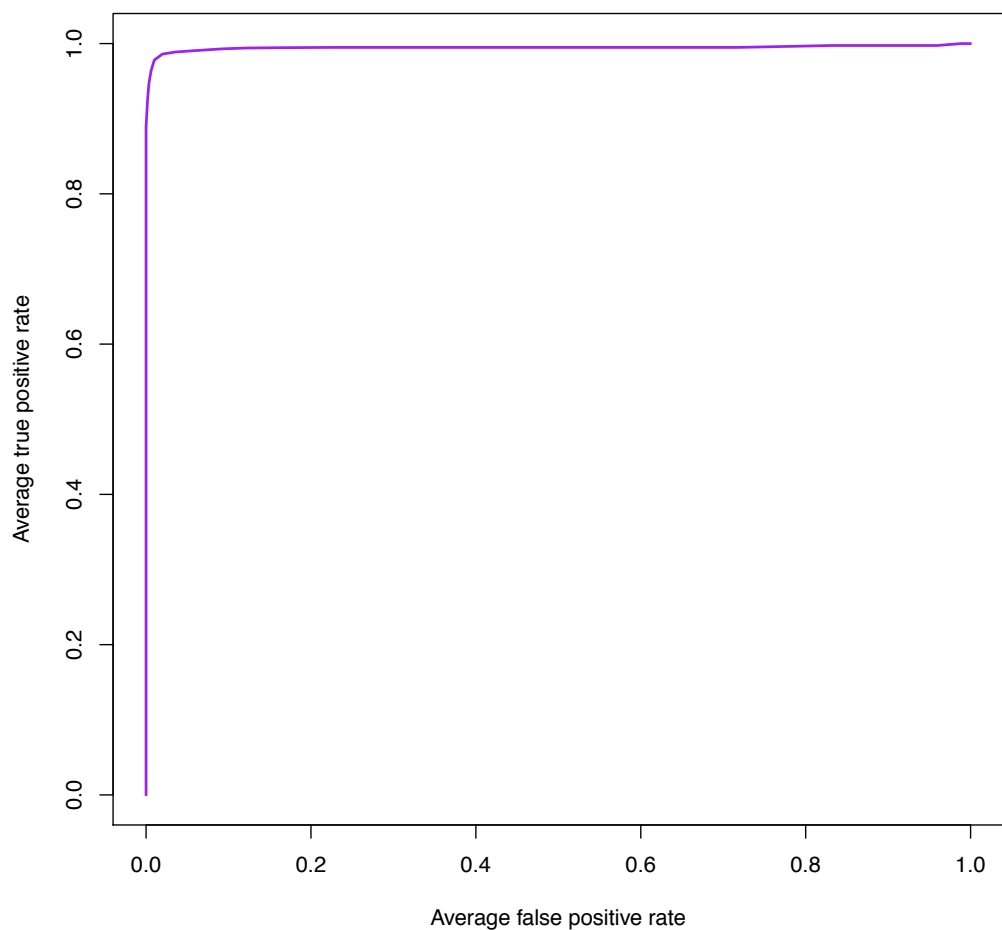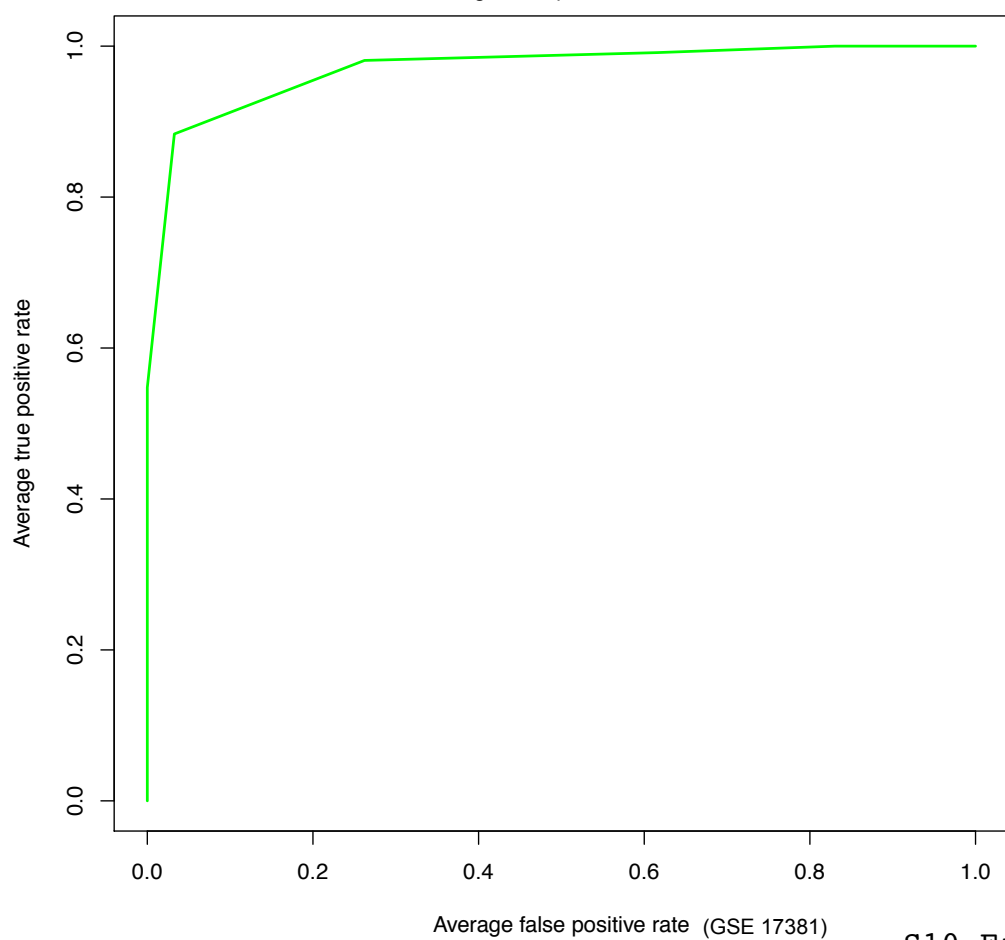

Supplement: S10 Fig — The purple line represents the classification performance ROC curve for expression profile data set GSE9891, whereas the green line represents that for expression profile data set GSE17308. (PDF) [file pone.0163353.s010.pdf]
