# Supplementary material for: A Systems Biology Comparison of Ovarian Cancers Implicates Putative Somatic Driver Mutations through Protein-Protein Interaction Models
Source: PLoS One. 2016 Oct 27;11(10):e0163353. doi: 10.1371/journal.pone.0163353 (PMC5082879; doi:10.1371/journal.pone.0163353)
Supplement: S8 Fig — (PDF) [file pone.0163353.s008.pdf]

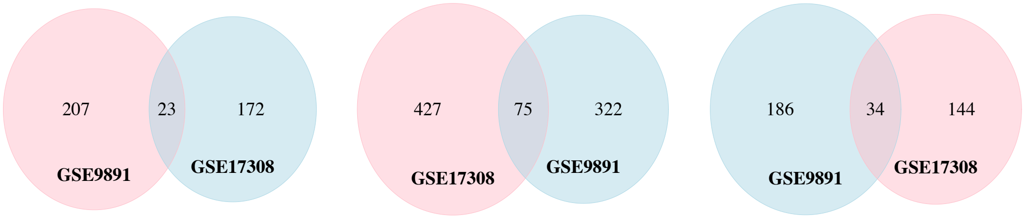

	GSE17308	GSE9891
**Differentially expressed genes**	195	230
**Subnetworks***	175 (502)	179 (397)
**Hub proteins**	178	220

*The number inside the parentheses is the number of genes that compose the corresponding subnetworks

S8 Fig
