# Supplementary material for: A Systems Biology Comparison of Ovarian Cancers Implicates Putative Somatic Driver Mutations through Protein-Protein Interaction Models
Source: PLoS One. 2016 Oct 27;11(10):e0163353. doi: 10.1371/journal.pone.0163353 (PMC5082879; doi:10.1371/journal.pone.0163353)
Supplement: S1 Table — (DOCX) [file pone.0163353.s011.docx]

**S1 Table. The significant subnetworks**

| SeedName | NodeNames | numNode | NodeLabe | ConnectNode |
| --- | --- | --- | --- | --- |
| AARS | AARS;YWHAZ;CHEK1 | 3 | 1;2;3 | 0;1;2 |
| ABL2 | ABL2;GRB2;DAG1;RACGAP1 | 4 | 1;2;3;4 | 0;1;2;2 |
| ADA | ADA;GRB2;DAG1;SUV39H2 | 4 | 1;2;3;4 | 0;1;2;2 |
| ADCK1 | ADCK1;RAB1A;GRB2;RACGAP1;PRC1 | 5 | 1;2;3;4;5 | 0;1;2;3;4 |
| ADCY3 | ADCY3;CAMK2G;RYR1;GRB2;RACGAP1;DAG1 | 6 | 1;2;3;4;5;6 | 0;1;2;3;4;4 |
| ADNP | ADNP;PLCG1;PTK2;STAT1;GRB2;LTK | 6 | 1;2;3;4;5;6 | 0;1;2;3;2;2 |
| AKAP6 | AKAP6;GRB2;RACGAP1;OCRL | 4 | 1;2;3;4 | 0;1;2;2 |
| ANGPT2 | ANGPT2;TEK;GRB2;RACGAP1;DAG1 | 5 | 1;2;3;4;5 | 0;1;2;3;3 |
| APLP1 | APLP1;TK1;YWHAZ;RFC4;AARS | 5 | 1;2;3;4;5 | 0;1;2;3;3 |
| APOF | APOF;APOA1;IGHM;PTK2;GRB2;ECT2 | 6 | 1;2;3;4;5;6 | 0;1;2;3;4;5 |
| ATF6 | ATF6;CREB1;ZNF35;MAPK14;EIF4EBP1;CDC25C | 6 | 1;2;3;4;5;6 | 0;1;2;2;4;4 |
| ATP6V0C | ATP6V0C;ARF6;MSH2;BLM;BARD1 | 5 | 1;2;3;4;5 | 0;1;2;3;3 |
| ATP6V1C1 | ATP6V1C1;ARF6;MSH2;CHEK1 | 4 | 1;2;3;4 | 0;1;2;3 |
| BATF | BATF;DDIT3;MAPK14;GMFB;CDC25A | 5 | 1;2;3;4;5 | 0;1;2;3;3 |
| BRD4 | BRD4;RFC4;YWHAZ;PCNA | 4 | 1;2;3;4 | 0;1;2;2 |
| BRI3BP | BRI3BP;TRAF6;MAPRE1;KIF2C;GNAI3 | 5 | 1;2;3;4;5 | 0;1;2;3;2 |
| CAD | CAD;GRB2;PTK2 | 3 | 1;2;3 | 0;1;2 |
| CCL4 | CCL4;CCBP2;EPB41;MAPRE1;RAB10 | 5 | 1;2;3;4;5 | 0;1;2;3;3 |
| CCNE2 | CCNE2;CDK2;SORT1 | 3 | 1;2;3 | 0;1;2 |
| CCR1 | CCR1;STAT1;MCM3;MCM2;GTF2I | 5 | 1;2;3;4;5 | 0;1;2;3;2 |
| CCT3 | CCT3;YWHAZ;CHEK1;PSMD2 | 4 | 1;2;3;4 | 0;1;2;2 |
| CCT4 | CCT4;CCNE1;MYBL2;YWHAZ;RFC4 | 5 | 1;2;3;4;5 | 0;1;2;1;4 |
| CCT5 | CCT5;H2AFX;CFL1;ILF2;MCM2 | 5 | 1;2;3;4;5 | 0;1;2;2;4 |
| CD19 | CD19;GRB2;RACGAP1;DAG1 | 4 | 1;2;3;4 | 0;1;2;2 |
| CD28 | CD28;GRB2;RACGAP1;DAG1 | 4 | 1;2;3;4 | 0;1;2;2 |
| CD33 | CD33;PTPN11;PTK2;GRB2;RACGAP1 | 5 | 1;2;3;4;5 | 0;1;2;2;4 |
| CD36 | CD36;SRC;CDC25C;CHEK2;MSH2 | 5 | 1;2;3;4;5 | 0;1;2;3;4 |
| CD72 | CD72;GRB2;RACGAP1;DAG1 | 4 | 1;2;3;4 | 0;1;2;2 |
| CHRD | CHRD;GRB2;RACGAP1;PTK2;EPHB1 | 5 | 1;2;3;4;5 | 0;1;2;2;2 |
| COBRA1 | COBRA1;GRB2;RACGAP1;DAG1;PTK2 | 5 | 1;2;3;4;5 | 0;1;2;2;2 |
| COL18A1 | COL18A1;GPC4;KDR;ACP1;SH2D2A;GRB2 | 6 | 1;2;3;4;5;6 | 0;1;1;3;3;3 |
| COX11 | COX11;SQSTM1;CHEK1;TIMELESS;MARK2 | 5 | 1;2;3;4;5 | 0;1;2;3;2 |
| CPS1 | CPS1;H2AFX;ILF2;MCM2;RPN2 | 5 | 1;2;3;4;5 | 0;1;2;3;2 |
| CSF1R | CSF1R;GRB2;RACGAP1;DAG1;PTK2 | 5 | 1;2;3;4;5 | 0;1;2;2;2 |
| CSF2RB | CSF2RB;YWHAZ;RFC4;CHEK1 | 4 | 1;2;3;4 | 0;1;2;2 |
| CSF3R | CSF3R;GRB2;RACGAP1;DAG1 | 4 | 1;2;3;4 | 0;1;2;2 |
| CUL1 | CUL1;CHEK1;CDCA3;E2F1 | 4 | 1;2;3;4 | 0;1;1;1 |
| CYFIP1 | CYFIP1;YWHAZ;RFC4;CHEK1 | 4 | 1;2;3;4 | 0;1;2;2 |
| DAG1 | DAG1;GRB2;RACGAP1 | 3 | 1;2;3 | 0;1;2 |
| DAXX | DAXX;MX1;BLM;CHEK1;TRPC4 | 5 | 1;2;3;4;5 | 0;1;2;3;2 |
| DDIT3 | DDIT3;HOXA5;CSNK2A1;CHEK1 | 4 | 1;2;3;4 | 0;1;1;3 |
| DDX54 | DDX54;ESR1;NR2F1;RPS15A | 4 | 1;2;3;4 | 0;1;2;2 |
| DLX2 | DLX2;NCOA2;PPARD;CDK4;ASPM | 5 | 1;2;3;4;5 | 0;1;2;2;4 |
| DLX4 | DLX4;GRB2;RACGAP1;DAG1 | 4 | 1;2;3;4 | 0;1;2;2 |
| DNMT1 | DNMT1;DNMT3B;YWHAZ;CHEK1 | 4 | 1;2;3;4 | 0;1;1;3 |
| DNTT | DNTT;PCNA;RFC4;POLQ | 4 | 1;2;3;4 | 0;1;2;2 |
| DOCK4 | DOCK4;YWHAB;CHEK1;RACGAP1;SRPK1;PAK4 | 6 | 1;2;3;4;5;6 | 0;1;2;2;2;2 |
| DSCAM | DSCAM;PAK1;MYNN;GRB2;RACGAP1;KIF23 | 6 | 1;2;3;4;5;6 | 0;1;2;2;4;5 |
| DUSP16 | DUSP16;MAPK14;CDC25A;CDKN3 | 4 | 1;2;3;4 | 0;1;2;3 |
| ECHS1 | ECHS1;YWHAZ;RFC4;CHEK1 | 4 | 1;2;3;4 | 0;1;2;2 |
| EEF2K | EEF2K;EEF2;DISC1;SYNE1 | 4 | 1;2;3;4 | 0;1;2;3 |
| EGF | EGF;GRB2;RACGAP1;DAG1 | 4 | 1;2;3;4 | 0;1;2;2 |
| EIF2B5 | EIF2B5;CSNK2A1;GMFB;DDIT3 | 4 | 1;2;3;4 | 0;1;2;2 |
| EMILIN3 | EMILIN3;SQSTM1;CHEK1;TIMELESS | 4 | 1;2;3;4 | 0;1;2;3 |
| EN1 | EN1;JUN;PRKDC;CHEK1;SPI1 | 5 | 1;2;3;4;5 | 0;1;2;3;2 |
| EPB41L1 | EPB41L1;YWHAZ;CHEK1;TIMELESS | 4 | 1;2;3;4 | 0;1;2;3 |
| EPPK1 | EPPK1;GRB2;RACGAP1;OCRL | 4 | 1;2;3;4 | 0;1;2;2 |
| ERO1L | ERO1L;H2AFX;ILF2;MCM2;CFL1 | 5 | 1;2;3;4;5 | 0;1;2;3;2 |
| F3 | F3;F10;SERPINB6;FYN;PTPRE;SLC24A1 | 6 | 1;2;3;4;5;6 | 0;1;2;2;4;4 |
| FBXO25 | FBXO25;CUL1;CHEK1;CDCA3;YWHAZ | 5 | 1;2;3;4;5 | 0;1;2;2;3 |
| FGF1 | FGF1;CSNK2A1;CHEK1;BLM | 4 | 1;2;3;4 | 0;1;2;3 |
| FIBP | FIBP;CSNK2A1;CHEK1;TIMELESS | 4 | 1;2;3;4 | 0;1;2;3 |
| FLT3 | FLT3;GRB2;RACGAP1;DAG1 | 4 | 1;2;3;4 | 0;1;2;2 |
| FTSJ3 | FTSJ3;GABARAP;HNRPDL;RPS9 | 4 | 1;2;3;4 | 0;1;2;2 |
| FUS | FUS;GRB2;RACGAP1;PTK2 | 4 | 1;2;3;4 | 0;1;2;2 |
| FYCO1 | FYCO1;SEC24B;GABARAP;RPL3 | 4 | 1;2;3;4 | 0;1;2;3 |
| GJA5 | GJA5;C20orf4;ARF6;MSH2;EXO1 | 5 | 1;2;3;4;5 | 0;1;2;3;4 |
| GNAZ | GNAZ;RGS19;CSNK2A1;CHEK1 | 4 | 1;2;3;4 | 0;1;2;3 |
| GNG3 | GNG3;GNAI3;RGS19;CSNK2A1;CHEK1;MSH2 | 6 | 1;2;3;4;5;6 | 0;1;2;3;4;5 |
| GRIN2B | GRIN2B;AP2M1;GRB2;RACGAP1;PTK2 | 5 | 1;2;3;4;5 | 0;1;2;3;3 |
| GTF3A | GTF3A;FYN;CAST;CNTN1;PLD2 | 5 | 1;2;3;4;5 | 0;1;2;2;2 |
| HAP1 | HAP1;GLTSCR2;KIAA1377;PCM1 | 4 | 1;2;3;4 | 0;1;1;1 |
| HBP1 | HBP1;ADRM1;PSMD2;UCHL5 | 4 | 1;2;3;4 | 0;1;2;2 |
| HDAC10 | HDAC10;NCOR2;STAT5B;THRA;NRIP1;RORB | 6 | 1;2;3;4;5;6 | 0;1;2;2;4;5 |
| HIST1H2BO | HIST1H2BO;ARRB1;KIF2C;ARPC5 | 4 | 1;2;3;4 | 0;1;2;2 |
| HMGA1 | HMGA1;ATF2;CEBPA;CDKN3;JUN;PRKDC | 6 | 1;2;3;4;5;6 | 0;1;2;3;1;5 |
| HOXD8 | HOXD8;HMGB1;AES;SNCAIP;GABARAP;RPS9 | 6 | 1;2;3;4;5;6 | 0;1;2;3;3;5 |
| HRC | HRC;TRDN;RYR1;NCK1;ECT2;RACGAP1 | 6 | 1;2;3;4;5;6 | 0;1;2;3;4;5 |
| HSPB2 | HSPB2;UBL5;CLK2;TOPBP1;PTPN1;PTK2 | 6 | 1;2;3;4;5;6 | 0;1;2;3;3;5 |
| HTR1A | HTR1A;GNAI3;RGS19;CSNK2A1;CHEK1;MSH2 | 6 | 1;2;3;4;5;6 | 0;1;2;3;4;5 |
| ICAM4 | ICAM4;ITGA4;PTK2;GRB2;RACGAP1 | 5 | 1;2;3;4;5 | 0;1;2;3;4 |
| INA | INA;ARRB1;KIF2C;YWHAZ | 4 | 1;2;3;4 | 0;1;2;2 |
| INPP5A | INPP5A;YWHAZ;RFC4;CAD | 4 | 1;2;3;4 | 0;1;2;2 |
| INPPL1 | INPPL1;GRB2;RACGAP1;PTK2 | 4 | 1;2;3;4 | 0;1;2;2 |
| IRAK1 | IRAK1;MAPK14;MARS;GMFB | 4 | 1;2;3;4 | 0;1;2;2 |
| IRAK1BP1 | IRAK1BP1;IRAK1;MAPK14;CDC25A;CHEK1 | 5 | 1;2;3;4;5 | 0;1;2;3;4 |
| JUB | JUB;GRB2;RACGAP1;ASXL1 | 4 | 1;2;3;4 | 0;1;2;2 |
| JUN | JUN;PRKDC;CHEK1;SPI1 | 4 | 1;2;3;4 | 0;1;2;1 |
| KCND1 | KCND1;IL16;CSNK2A1;CHEK1;TIMELESS | 5 | 1;2;3;4;5 | 0;1;2;3;4 |
| KCNJ1 | KCNJ1;PRKCD;PIK3CA;NRAS;RACGAP1 | 5 | 1;2;3;4;5 | 0;1;2;3;4 |
| KRT4 | KRT4;UCHL1;COPS5;UBE2H;PPIL5 | 5 | 1;2;3;4;5 | 0;1;2;3;3 |
| LACTB | LACTB;ESR1;MKI67;FKBP4;TUBB;DEK | 6 | 1;2;3;4;5;6 | 0;1;2;2;2;2 |
| LAMP1 | LAMP1;AP2M1;GRB2;RACGAP1;KIF23 | 5 | 1;2;3;4;5 | 0;1;2;3;4 |
| MAP1LC3A | MAP1LC3A;GMPS;MCM3;MCM6 | 4 | 1;2;3;4 | 0;1;1;3 |
| MAP2 | MAP2;GRB2;RACGAP1;FOXH1 | 4 | 1;2;3;4 | 0;1;2;2 |
| MAP2K5 | MAP2K5;MAP3K3;ST13;TNFRSF14;GABARAPL2;EEF2 | 6 | 1;2;3;4;5;6 | 0;1;2;3;3;5 |
| MAP2K6 | MAP2K6;MAPK14;CDC25C;CHEK1 | 4 | 1;2;3;4 | 0;1;2;3 |
| MAP3K10 | MAP3K10;RACGAP1;NRAS | 3 | 1;2;3 | 0;1;2 |
| MAP3K5 | MAP3K5;CDKN1A;QARS;DMD | 4 | 1;2;3;4 | 0;1;1;1 |
| MAP3K7IP2 | MAP3K7IP2;IRAK1;BTK;DBN1;TEC;PRKCE;COPB2 | 7 | 1;2;3;4;5;6;7 | 0;1;2;3;3;3;6 |
| MAPK12 | MAPK12;GRB2;RACGAP1;OCRL | 4 | 1;2;3;4 | 0;1;2;2 |
| MAPK4 | MAPK4;GAB2;GRB2;RACGAP1;PRC1 | 5 | 1;2;3;4;5 | 0;1;2;3;4 |
| MAPK8 | MAPK8;PRKDC;CHEK1;NCF4 | 4 | 1;2;3;4 | 0;1;2;2 |
| MBTPS1 | MBTPS1;BDNF;ESR1;RPS20;RPL3 | 5 | 1;2;3;4;5 | 0;1;2;3;3 |
| MCM4 | MCM4;ILF2;MCM6 | 3 | 1;2;3 | 0;1;1 |
| MEPE | MEPE;GRB2;RACGAP1;FOXH1 | 4 | 1;2;3;4 | 0;1;2;2 |
| MMP13 | MMP13;TIMP3;KDR;ACP1;GRB2;PTK2 | 6 | 1;2;3;4;5;6 | 0;1;2;3;3;5 |
| MMP14 | MMP14;TIMP3;KDR;ACP1;STAT1;PTK2 | 6 | 1;2;3;4;5;6 | 0;1;2;3;3;5 |
| MNDA | MNDA;CDK2;BIRC5;CDKN3 | 4 | 1;2;3;4 | 0;1;2;2 |
| NAGPA | NAGPA;AP2A1;GRB2;RACGAP1;KIF23;NEU3 | 6 | 1;2;3;4;5;6 | 0;1;2;3;4;3 |
| NCK2 | NCK2;PTK2;GRB2;RACGAP1;HIPK3 | 5 | 1;2;3;4;5 | 0;1;2;3;3 |
| NDUFA9 | NDUFA9;CIAO1;MCM3;YWHAZ | 4 | 1;2;3;4 | 0;1;2;3 |
| NEU3 | NEU3;GRB2;RACGAP1;KIF23 | 4 | 1;2;3;4 | 0;1;2;3 |
| NFS1 | NFS1;GRB2;RACGAP1 | 3 | 1;2;3 | 0;1;2 |
| NOXA1 | NOXA1;YWHAZ;CHEK1;TIMELESS;PSMD1 | 5 | 1;2;3;4;5 | 0;1;2;3;2 |
| NQO1 | NQO1;DNAJB1;FANCA;GRB2;RACGAP1 | 5 | 1;2;3;4;5 | 0;1;2;3;4 |
| NSD1 | NSD1;RELA;CHEK1;TIMELESS | 4 | 1;2;3;4 | 0;1;2;3 |
| NTS | NTS;SORT1;CDK2;CCNA2 | 4 | 1;2;3;4 | 0;1;2;3 |
| NUDT5 | NUDT5;TRAF6;MCM3;RAB10 | 4 | 1;2;3;4 | 0;1;2;2 |
| OASL | OASL;MBD1;PCNA;CHEK1;BLM | 5 | 1;2;3;4;5 | 0;1;2;3;4 |
| PANK2 | PANK2;YWHAZ;KIF23;CIC | 4 | 1;2;3;4 | 0;1;2;2 |
| PANX3 | PANX3;BCL6;SPTLC2;BCL6B;MTA3;GRB2;RACGAP1 | 7 | 1;2;3;4;5;6;7 | 0;1;2;2;2;5;6 |
| PARVA | PARVA;SNW1;EEF1A1;GABARAP;RPL12 | 5 | 1;2;3;4;5 | 0;1;2;3;2 |
| PCBP4 | PCBP4;PCBP1;YWHAZ;RFC4;CHEK1 | 5 | 1;2;3;4;5 | 0;1;2;3;3 |
| PDE3B | PDE3B;YWHAB;CDC25A;SRPK1;KIF23 | 5 | 1;2;3;4;5 | 0;1;2;2;2 |
| PECR | PECR;ISL1;NR2F1;ESR1;CDKN1A;RPS15A;RPL3 | 7 | 1;2;3;4;5;6;7 | 0;1;2;2;4;4;4 |
| PES1 | PES1;ARRB1;KIF2C;ARPC5;NOLC1 | 5 | 1;2;3;4;5 | 0;1;2;2;2 |
| PEX13 | PEX13;GRB2;RACGAP1;FOXH1 | 4 | 1;2;3;4 | 0;1;2;2 |
| PEX26 | PEX26;SUFU;SETDB1;TK1;YWHAZ;AARS | 6 | 1;2;3;4;5;6 | 0;1;2;3;4;5 |
| PKLR | PKLR;KIF23;YWHAZ;RFC4 | 4 | 1;2;3;4 | 0;1;2;3 |
| PLEC1 | PLEC1;LMNB1;MCM10;MCM6 | 4 | 1;2;3;4 | 0;1;2;3 |
| PPARD | PPARD;NCOA2;PPFIA1;CDK4;PCNA | 5 | 1;2;3;4;5 | 0;1;2;2;4 |
| PPP1R13B | PPP1R13B;TP73;CCNG1;DAB2IP;MAP3K5;CDKN1A;HMGB1 | 7 | 1;2;3;4;5;6;7 | 0;1;2;2;4;5;2 |
| PRKAR2A | PRKAR2A;ARFGEF2;AKAP8;MCM2;MCM6 | 5 | 1;2;3;4;5 | 0;1;1;3;4 |
| PRPF31 | PRPF31;FANCA;GRB2;RACGAP1;DAG1 | 5 | 1;2;3;4;5 | 0;1;2;3;3 |
| PSMB5 | PSMB5;UCHL5;PSMD2;YWHAZ;CDC25A | 5 | 1;2;3;4;5 | 0;1;2;3;4 |
| PSMD7 | PSMD7;PSMD2;UCHL5;YWHAZ;TUBB | 5 | 1;2;3;4;5 | 0;1;1;2;4 |
| PTEN | PTEN;ANAPC4;CSNK2A2;PRKCA;SLC4A4 | 5 | 1;2;3;4;5 | 0;1;1;1;4 |
| PTPN22 | PTPN22;GRB2;RACGAP1;DAG1 | 4 | 1;2;3;4 | 0;1;2;2 |
| PYGO1 | PYGO1;SPTBN1;STAT1;PTK2;CSE1L | 5 | 1;2;3;4;5 | 0;1;2;3;3 |
| RANBP2 | RANBP2;GABARAPL2;DNAH7;ST13 | 4 | 1;2;3;4 | 0;1;2;2 |
| RPP38 | RPP38;GRB2;RACGAP1;CAD | 4 | 1;2;3;4 | 0;1;2;2 |
| RYR1 | RYR1;GRB2;RACGAP1;DAG1 | 4 | 1;2;3;4 | 0;1;2;2 |
| SERP1 | SERP1;DNAJB1;FANCA;GRB2;RACGAP1 | 5 | 1;2;3;4;5 | 0;1;2;3;4 |
| SGCA | SGCA;DAG1;GRB2;RACGAP1;PRC1 | 5 | 1;2;3;4;5 | 0;1;2;3;4 |
| SGCZ | SGCZ;DMD;MAP3K5;CDKN1A;QARS;TP53 | 6 | 1;2;3;4;5;6 | 0;1;2;3;3;4 |
| SLIT3 | SLIT3;CAPN1;ARF6;MSH2;PSMD8 | 5 | 1;2;3;4;5 | 0;1;2;3;3 |
| SNX7 | SNX7;GRB2;RACGAP1;DAG1;PTK2 | 5 | 1;2;3;4;5 | 0;1;2;2;2 |
| SOS2 | SOS2;GRB2;RACGAP1;PTK2 | 4 | 1;2;3;4 | 0;1;2;2 |
| SPAG1 | SPAG1;PRKCA;GPM6A;RASGRF1;KRT18 | 5 | 1;2;3;4;5 | 0;1;2;2;2 |
| SPTAN1 | SPTAN1;GABARAP;EEF1A1;RPS9 | 4 | 1;2;3;4 | 0;1;2;2 |
| SRF | SRF;PRKDC;CHEK1;CDC25B;SQSTM1 | 5 | 1;2;3;4;5 | 0;1;2;3;3 |
| SRPX | SRPX;MAP1LC3A;EEF1A1;RPL3;RPS9 | 5 | 1;2;3;4;5 | 0;1;2;2;2 |
| SS18 | SS18;GRB2;RACGAP1;PTK2 | 4 | 1;2;3;4 | 0;1;2;2 |
| STRBP | STRBP;GABARAPL2;EEF1A1;HNRPDL | 4 | 1;2;3;4 | 0;1;2;2 |
| STX16 | STX16;VAMP4;CSNK2A1;CHEK1;BLM | 5 | 1;2;3;4;5 | 0;1;2;3;4 |
| SYP | SYP;GRB2;RACGAP1;PTK2 | 4 | 1;2;3;4 | 0;1;2;2 |
| TAF4 | TAF4;CREB1;RPS6KA4;TAF11 | 4 | 1;2;3;4 | 0;1;2;1 |
| TAF5L | TAF5L;CEBPE;DDIT3;HOXA5;CSNK2A1 | 5 | 1;2;3;4;5 | 0;1;2;3;3 |
| TAF6L | TAF6L;CREBBP;FOXM1;CDC20;MAML1 | 5 | 1;2;3;4;5 | 0;1;2;2;2 |
| TBC1D5 | TBC1D5;AP2M1;GRB2;RACGAP1;PRC1 | 5 | 1;2;3;4;5 | 0;1;2;3;4 |
| TFAP2B | TFAP2B;GRB2;RACGAP1;NFS1 | 4 | 1;2;3;4 | 0;1;2;2 |
| TFPI2 | TFPI2;SKIL;TTF2;MYBL2;DRAP1;SKP2 | 6 | 1;2;3;4;5;6 | 0;1;2;3;3;4 |
| TGFB1I1 | TGFB1I1;PTK2;STAT1;MCM3;GTF2I | 5 | 1;2;3;4;5 | 0;1;2;3;3 |
| TGM1 | TGM1;GABARAPL2;EEF1A1;DNAH7;TTBK2 | 5 | 1;2;3;4;5 | 0;1;2;2;2 |
| TGOLN2 | TGOLN2;GRB2;RACGAP1;DAG1 | 4 | 1;2;3;4 | 0;1;2;2 |
| TNFRSF1B | TNFRSF1B;PRKDC;CHEK1;XPOT | 4 | 1;2;3;4 | 0;1;2;1 |
| TRIM39 | TRIM39;GRB2;RACGAP1;DAG1 | 4 | 1;2;3;4 | 0;1;2;2 |
| TRIO | TRIO;PTK2;GRB2;CAD;DAG1 | 5 | 1;2;3;4;5 | 0;1;2;3;3 |
| TRPC4 | TRPC4;MX1;BLM;CHEK1 | 4 | 1;2;3;4 | 0;1;2;3 |
| TRPM6 | TRPM6;MYH9;PRKCE;GRB2;RACGAP1;DAG1 | 6 | 1;2;3;4;5;6 | 0;1;2;2;4;4 |
| USF1 | USF1;PRKDC;CHEK1;TIMELESS | 4 | 1;2;3;4 | 0;1;2;3 |
| VCL | VCL;PTK2;YWHAZ;RFC4;STAT1 | 5 | 1;2;3;4;5 | 0;1;1;3;2 |
| WDR18 | WDR18;ESR1;CDKN1A;RPL3 | 4 | 1;2;3;4 | 0;1;2;2 |
| ZNF426 | ZNF426;LPXN;PTK2;GRB2;DAG1;CAD | 6 | 1;2;3;4;5;6 | 0;1;2;3;4;4 |
| ADA | ADA;GRB2;ABL1;PDCD6IP;SOS2 | 5 | 1;2;3;4;5 | 0;1;2;2;2 |
| ADAM11 | ADAM11;STC2;ARRB2;RALGDS;ERH;PIK3C2A | 6 | 1;2;3;4;5;6 | 0;1;2;3;3;3 |
| AGR2 | AGR2;CRMP1;VIM;MRPL44 | 4 | 1;2;3;4 | 0;1;2;3 |
| ALCAM | ALCAM;EGFR;EPHA2;CBLB;PRKCD | 5 | 1;2;3;4;5 | 0;1;2;2;2 |
| ANKRA2 | ANKRA2;HDAC5;ESR1;MARK3 | 4 | 1;2;3;4 | 0;1;2;2 |
| AP2S1 | AP2S1;AP1G1;RABGEF1;RNF11;MYO6 | 5 | 1;2;3;4;5 | 0;1;2;3;4 |
| APBB3 | APBB3;APLP2;PRNP;CLSTN1;PLG | 5 | 1;2;3;4;5 | 0;1;2;3;3 |
| ARHGEF11 | ARHGEF11;PLXNB2;ABL1;EFNA5;NBEA | 5 | 1;2;3;4;5 | 0;1;1;3;3 |
| ATRX | ATRX;SVIL;MYH10;NR3C1;BAG1 | 5 | 1;2;3;4;5 | 0;1;2;2;4 |
| B4GALT6 | B4GALT6;GOLGA2;STK25;YWHAZ;PPP2R1B | 5 | 1;2;3;4;5 | 0;1;2;3;3 |
| BANP | BANP;MDM2;PIAS1;GATA4;S100A6 | 5 | 1;2;3;4;5 | 0;1;2;3;2 |
| BCAN | BCAN;ADAMTS4;FN1;LTBP1;COL4A4 | 5 | 1;2;3;4;5 | 0;1;2;3;3 |
| BCL2L13 | BCL2L13;GABARAPL2;DNAH7;VDAC1;GABARAPL1 | 5 | 1;2;3;4;5 | 0;1;2;2;2 |
| BLM | BLM;ATM;FEN1;DCLRE1A;TOPBP1 | 5 | 1;2;3;4;5 | 0;1;1;2;2 |
| BTK | BTK;SYK;GRB2;OCRL;DAAM1 | 5 | 1;2;3;4;5 | 0;1;1;3;1 |
| BTRC | BTRC;CTNNB1;PSEN2 | 3 | 1;2;3 | 0;1;2 |
| C21orf63 | C21orf63;RBBP6;RPS14;MAP3K1;MDM2 | 5 | 1;2;3;4;5 | 0;1;2;3;2 |
| C6orf48 | C6orf48;CFTR;IPO7;CD59 | 4 | 1;2;3;4 | 0;1;2;2 |
| CAMKK2 | CAMKK2;IQGAP2;PRKAA2;MYC;COPG2;HADHB | 6 | 1;2;3;4;5;6 | 0;1;1;2;4;4 |
| CAV1 | CAV1;CTNNB1;CDC27;TRAF2;BTRC | 5 | 1;2;3;4;5 | 0;1;2;1;2 |
| CBR1 | CBR1;GABARAPL2;RPL15;GUCY2F | 4 | 1;2;3;4 | 0;1;2;2 |
| CCL18 | CCL18;CRMP1;AGR2;PPP1R8;VIM | 5 | 1;2;3;4;5 | 0;1;2;2;2 |
| CCT4 | CCT4;MAP3K1;VIM;MYH10;RPS9 | 5 | 1;2;3;4;5 | 0;1;2;2;2 |
| CD63 | CD63;CD9;CD59;GRB2;CD2 | 5 | 1;2;3;4;5 | 0;1;2;3;3 |
| CDC25A | CDC25A;CHEK1;BTRC;SFN | 4 | 1;2;3;4 | 0;1;1;2 |
| CDC25B | CDC25B;CHEK1;XRCC5;HSF1 | 4 | 1;2;3;4 | 0;1;2;3 |
| CDC45L | CDC45L;CDKN1A;CHEK1;BCCIP | 4 | 1;2;3;4 | 0;1;2;2 |
| CDH1 | CDH1;CAV1;GJB1;MDM2 | 4 | 1;2;3;4 | 0;1;2;1 |
| CDH10 | CDH10;CTNNB1;BTRC;ESR1;RPL12 | 5 | 1;2;3;4;5 | 0;1;2;2;4 |
| CDH8 | CDH8;CTNNB1;BTRC;PSEN2 | 4 | 1;2;3;4 | 0;1;2;2 |
| CDKL5 | CDKL5;GRB2;PAK4;SYK;DARS | 5 | 1;2;3;4;5 | 0;1;2;2;2 |
| CDKN2B | CDKN2B;IKBKAP;MYC;SP1;SMARCAD1 | 5 | 1;2;3;4;5 | 0;1;2;3;3 |
| CENPA | CENPA;DIAPH1;CDC7;CDKN2A;CDK4 | 5 | 1;2;3;4;5 | 0;1;2;3;3 |
| CHRM4 | CHRM4;GRB2;PPP3CA;ANK2 | 4 | 1;2;3;4 | 0;1;2;2 |
| CHUK | CHUK;CHEK1;IRAK1;CTNNB1 | 4 | 1;2;3;4 | 0;1;1;1 |
| COX17 | COX17;TP53;PTEN | 3 | 1;2;3 | 0;1;2 |
| CPSF2 | CPSF2;WWOX;TP73;YAP1;TEAD1 | 5 | 1;2;3;4;5 | 0;1;2;3;4 |
| CSN2 | CSN2;GRB2;PPP3CA;ANK2;DNM2 | 5 | 1;2;3;4;5 | 0;1;2;2;2 |
| CSNK1D | CSNK1D;PER1;BTRC;MDM2;S100A2 | 5 | 1;2;3;4;5 | 0;1;2;1;4 |
| CTSB | CTSB;ANXA2;ARRB1;DNAH3;NDUFS1 | 5 | 1;2;3;4;5 | 0;1;2;3;3 |
| CXCR4 | CXCR4;STAT1;SDC4 | 3 | 1;2;3 | 0;1;1 |
| DAP3 | DAP3;GABARAP;SRRM2;CNN3 | 4 | 1;2;3;4 | 0;1;2;2 |
| DCLRE1C | DCLRE1C;BRCA1;SMARCD2;NPM1;TAF9 | 5 | 1;2;3;4;5 | 0;1;2;2;2 |
| DEFB1 | DEFB1;VIM;MAP3K1;PPL | 4 | 1;2;3;4 | 0;1;2;2 |
| DLX2 | DLX2;HOXC8;FYN;CDH1;AKAP6 | 5 | 1;2;3;4;5 | 0;1;2;3;3 |
| DNAJA2 | DNAJA2;MAP1LC3B;EEF2;PRDX3 | 4 | 1;2;3;4 | 0;1;2;2 |
| DNAJC3 | DNAJC3;HSPA8;ESR1;XRCC5;CHEK1 | 5 | 1;2;3;4;5 | 0;1;2;3;4 |
| DNALI1 | DNALI1;EPS8;ITGB3;DOK1 | 4 | 1;2;3;4 | 0;1;2;3 |
| DOCK4 | DOCK4;YWHAB;MAP3K3;CDC25C | 4 | 1;2;3;4 | 0;1;2;2 |
| DOK1 | DOK1;ITGB3;ERBB2 | 3 | 1;2;3 | 0;1;1 |
| DTNBP1 | DTNBP1;PLCG1;DGKZ;MAP4 | 4 | 1;2;3;4 | 0;1;2;2 |
| DUSP14 | DUSP14;CD28;GRB2;CD22;SH3BP2 | 5 | 1;2;3;4;5 | 0;1;2;3;3 |
| EEF2K | EEF2K;RPS6KB1;CCNB1;MAP4 | 4 | 1;2;3;4 | 0;1;1;3 |
| EIF5A | EIF5A;TGM2;ITGB3;RGS12 | 4 | 1;2;3;4 | 0;1;2;3 |
| ENTPD5 | ENTPD5;PRKCA;PAM;SLC6A9;OCLN | 5 | 1;2;3;4;5 | 0;1;2;2;2 |
| EP400 | EP400;TRRAP;TP53;ZFP36L1;CREB1 | 5 | 1;2;3;4;5 | 0;1;2;3;3 |
| EPHB4 | EPHB4;GRIN1;PLAT;ANXA2;PLG | 5 | 1;2;3;4;5 | 0;1;2;3;3 |
| EPRS | EPRS;GABARAPL2;RPS23;GUCY2F;IKBKB | 5 | 1;2;3;4;5 | 0;1;2;2;1 |
| F2RL1 | F2RL1;F2;PLG;ANXA2;SERPINB13 | 5 | 1;2;3;4;5 | 0;1;1;3;3 |
| FGG | FGG;ITGB3;DOK1 | 3 | 1;2;3 | 0;1;2 |
| FLNB | FLNB;ITGB3;GRB2;RYR1 | 4 | 1;2;3;4 | 0;1;1;3 |
| FLNC | FLNC;PLCG1;PLXNB2;PICALM | 4 | 1;2;3;4 | 0;1;2;2 |
| FRAS1 | FRAS1;TP53;CHEK1;CCNG1;GTF2H1 | 5 | 1;2;3;4;5 | 0;1;2;2;2 |
| GABRB2 | GABRB2;PPP3CA;GRB2;CD22 | 4 | 1;2;3;4 | 0;1;2;3 |
| GAD1 | GAD1;GRB2;SPRY2;PRKAR1A;PPP3CA | 5 | 1;2;3;4;5 | 0;1;2;2;2 |
| GEMIN4 | GEMIN4;SPRY1;SNRPN;MAP3K1;RPS14 | 5 | 1;2;3;4;5 | 0;1;1;1;4 |
| GIPC2 | GIPC2;PTN;BCCIP;PPP2CA;CCNG1 | 5 | 1;2;3;4;5 | 0;1;2;2;4 |
| GLTSCR2 | GLTSCR2;APEX1;VIM;XRCC5 | 4 | 1;2;3;4 | 0;1;2;2 |
| GMNN | GMNN;CDKN1A;BCCIP;CHEK1;NEK9 | 5 | 1;2;3;4;5 | 0;1;2;2;4 |
| GPR25 | GPR25;MAP3K7;HSPA1L;NRIP1;PPARG | 5 | 1;2;3;4;5 | 0;1;2;2;4 |
| GPR37 | GPR37;DNAJB1;HSF1;XRCC5;PRKAA2 | 5 | 1;2;3;4;5 | 0;1;2;3;2 |
| GPT | GPT;CAPN1;F2RL1;SYNE1;ITGB3;VIM | 6 | 1;2;3;4;5;6 | 0;1;2;2;2;2 |
| GRB2 | GRB2;PPP3CA;SPRY2 | 3 | 1;2;3 | 0;1;1 |
| GUCY2F | GUCY2F;GABARAPL2;RPS23;PWP1 | 4 | 1;2;3;4 | 0;1;2;2 |
| HCN4 | HCN4;ABL1;JAK1;MAPT;SHB | 5 | 1;2;3;4;5 | 0;1;2;2;2 |
| HDLBP | HDLBP;DHX9;TBP;TAF11;BTAF1 | 5 | 1;2;3;4;5 | 0;1;2;3;3 |
| HIST1H2BO | HIST1H2BO;ARRB2;AP2M1;PHKA2;NDUFS7 | 5 | 1;2;3;4;5 | 0;1;2;2;2 |
| HMGCR | HMGCR;PRKCA;SLC6A9;ADD3 | 4 | 1;2;3;4 | 0;1;2;2 |
| HSF2 | HSF2;HSF1;TAF9;MYC | 4 | 1;2;3;4 | 0;1;2;3 |
| HTR1F | HTR1F;CAV1;EGFR;EPHA2;PRKAR1A | 5 | 1;2;3;4;5 | 0;1;2;3;3 |
| INA | INA;SQSTM1;NTRK3;GABARAP;TIAL1 | 5 | 1;2;3;4;5 | 0;1;2;2;4 |
| INPP4A | INPP4A;CAPN1;F2RL1;ITGB3;DOK1;INPP5D | 6 | 1;2;3;4;5;6 | 0;1;2;2;4;5 |
| ITGB1BP2 | ITGB1BP2;RARA;NPM1;GRB2;NPAS2 | 5 | 1;2;3;4;5 | 0;1;2;3;2 |
| ITM2B | ITM2B;BCL2;PPP3CA;GRB2;HOXC8 | 5 | 1;2;3;4;5 | 0;1;2;3;4 |
| KHDRBS1 | KHDRBS1;SNRPN;ESR1;RPL18 | 4 | 1;2;3;4 | 0;1;1;3 |
| KIAA0408 | KIAA0408;VIM;GABARAPL2;CCT2 | 4 | 1;2;3;4 | 0;1;2;3 |
| KLHDC2 | KLHDC2;SFN;SRRM2;SNRPF;PRNP | 5 | 1;2;3;4;5 | 0;1;2;3;3 |
| LETM1 | LETM1;MCC;TBCA;PDCD6IP | 4 | 1;2;3;4 | 0;1;2;2 |
| LGTN | LGTN;RAD51;BCCIP;VIM;CHEK1 | 5 | 1;2;3;4;5 | 0;1;2;2;2 |
| MAGED1 | MAGED1;COPG2;NEK6;SIRT7;SPTLC1 | 5 | 1;2;3;4;5 | 0;1;2;1;1 |
| MAP1LC3B | MAP1LC3B;EEF2;CLTC | 3 | 1;2;3 | 0;1;1 |
| MAPK8IP3 | MAPK8IP3;MAP3K1;VIM;RPS14 | 4 | 1;2;3;4 | 0;1;2;2 |
| MAPKAPK3 | MAPKAPK3;EEF2;PPP2R1A;MAP1LC3B;JUP | 5 | 1;2;3;4;5 | 0;1;2;2;4 |
| MARCKS | MARCKS;PRKCA;MYOD1;IFRD1;PPP1R8 | 5 | 1;2;3;4;5 | 0;1;2;3;2 |
| MLH3 | MLH3;MLH1;MYC;MYO5C;PMS1 | 5 | 1;2;3;4;5 | 0;1;2;3;2 |
| MPHOSPH9 | MPHOSPH9;YWHAZ;RPL31;VIM;KRT19 | 5 | 1;2;3;4;5 | 0;1;2;2;2 |
| MRPL44 | MRPL44;VIM;GABARAPL2;RPL15 | 4 | 1;2;3;4 | 0;1;2;3 |
| MRPS11 | MRPS11;MAP1LC3A;EEF2;NEDD8;NONO | 5 | 1;2;3;4;5 | 0;1;2;3;2 |
| MSI2 | MSI2;GRB2;ANXA2;MYLK | 4 | 1;2;3;4 | 0;1;2;2 |
| MTA1 | MTA1;ESR1;RPL15;NRIP1;GRB2 | 5 | 1;2;3;4;5 | 0;1;2;2;1 |
| MYBPC2 | MYBPC2;GRB2;PPP3CA;SPRY1;ERBB2 | 5 | 1;2;3;4;5 | 0;1;2;2;2 |
| MYO1E | MYO1E;ACTB;RPL18;MAP3K1;MAP4K2;RPS14 | 6 | 1;2;3;4;5;6 | 0;1;2;3;4;4 |
| MYO1G | MYO1G;TFRC;TRAF6;PSMC2;CTPS | 5 | 1;2;3;4;5 | 0;1;2;3;3 |
| NAP1L1 | NAP1L1;CREBBP;TCF7L1;MSH2;MYBL2;KHDRBS1 | 6 | 1;2;3;4;5;6 | 0;1;2;2;2;2 |
| NCALD | NCALD;TUBB;MAP3K1;HSPA1L | 4 | 1;2;3;4 | 0;1;2;3 |
| NEDD4L | NEDD4L;NR3C1;ARHGDIA;BAG1;NR2E3 | 5 | 1;2;3;4;5 | 0;1;1;2;2 |
| NEDD8 | NEDD8;EEF2;MAP1LC3B | 3 | 1;2;3 | 0;1;2 |
| NONO | NONO;RIPK3;MYH10;VIM;TRIM16 | 5 | 1;2;3;4;5 | 0;1;2;2;4 |
| NUFIP1 | NUFIP1;BRCA1;NPM1;STAT3 | 4 | 1;2;3;4 | 0;1;2;2 |
| OGG1 | OGG1;PRKCA;CHAT;BTK | 4 | 1;2;3;4 | 0;1;2;2 |
| OPRL1 | OPRL1;RGS19;TGFBR1;RAN | 4 | 1;2;3;4 | 0;1;2;3 |
| OR10J1 | OR10J1;TRAF6;TFRC;DNAJA1;IRAK3 | 5 | 1;2;3;4;5 | 0;1;2;2;2 |
| OSMR | OSMR;ERBB2;CTNNB1;DOK1;CDH17;MYO7A | 6 | 1;2;3;4;5;6 | 0;1;2;2;3;3 |
| PANX2 | PANX2;GRB2;PPP3CA;EPHA2;PTPN4 | 5 | 1;2;3;4;5 | 0;1;2;2;2 |
| PDGFRB | PDGFRB;STAT1;CXCR4;TYK2 | 4 | 1;2;3;4 | 0;1;2;1 |
| PDHX | PDHX;DLAT;MAP1LC3B;EEF2;GABARAP;PPP2CA | 6 | 1;2;3;4;5;6 | 0;1;2;3;3;4 |
| POLR2C | POLR2C;TAF15;GTF2H1;GTF2F2;SAFB;POLR2D | 6 | 1;2;3;4;5;6 | 0;1;1;1;2;1 |
| POU4F2 | POU4F2;ESR1;NRIP1;RPL15 | 4 | 1;2;3;4 | 0;1;2;2 |
| PPP1R12B | PPP1R12B;HSPA8;HSF1;BAG1;ENO2 | 5 | 1;2;3;4;5 | 0;1;2;2;3 |
| PRKAR1A | PRKAR1A;GRB2;CD22;PSTPIP2 | 4 | 1;2;3;4 | 0;1;2;2 |
| PRKCA | PRKCA;SLC6A9 | 2 | 1;2 | 0;1 |
| PRKCH | PRKCH;NUMB;ITGB3;DOK1;YES1 | 5 | 1;2;3;4;5 | 0;1;2;3;3 |
| PRKD2 | PRKD2;RARB;NCOA1;NR5A1;RBPMS | 5 | 1;2;3;4;5 | 0;1;2;3;4 |
| PRKX | PRKX;PRKAR1A;GRB2;CD22;LCP2 | 5 | 1;2;3;4;5 | 0;1;2;3;3 |
| PTPN4 | PTPN4;GRB2;NPM1;PPP3CA | 4 | 1;2;3;4 | 0;1;2;2 |
| PTPRB | PTPRB;PDGFRB;CAV1;ITGB3;GRB2 | 5 | 1;2;3;4;5 | 0;1;2;2;2 |
| PTPRU | PTPRU;PPFIA3;CAST;GRB2;TFAP2B | 5 | 1;2;3;4;5 | 0;1;2;3;4 |
| RAB25 | RAB25;SMURF2;SMURF1;TANK;HIVEP3 | 5 | 1;2;3;4;5 | 0;1;2;2;4 |
| RAD51 | RAD51;BCCIP;CHEK1;VIM | 4 | 1;2;3;4 | 0;1;1;1 |
| RAI2 | RAI2;CTBP2;NRIP1;NR3C1;BAG1;ST13 | 6 | 1;2;3;4;5;6 | 0;1;2;3;4;5 |
| RASA1 | RASA1;DOK1;ITGB3;HSPD1 | 4 | 1;2;3;4 | 0;1;2;1 |
| RASGRF1 | RASGRF1;RAC1;IQGAP1;CHN2 | 4 | 1;2;3;4 | 0;1;2;2 |
| RBM14 | RBM14;XRCC5;CHEK1;ORC1L;ABL1 | 5 | 1;2;3;4;5 | 0;1;2;2;4 |
| REPS1 | REPS1;HSF1;TAF9;GRB2;SNX17 | 5 | 1;2;3;4;5 | 0;1;2;1;4 |
| RFX4 | RFX4;ESR1;RPL7;HSPA8;MYC;RPL14 | 6 | 1;2;3;4;5;6 | 0;1;2;2;2;2 |
| RIMS2 | RIMS2;GRB2;SPRY1;CDC27 | 4 | 1;2;3;4 | 0;1;2;2 |
| RNGTT | RNGTT;MYC;MYO5C;BTAF1 | 4 | 1;2;3;4 | 0;1;2;2 |
| RPL10L | RPL10L;GABARAPL2;RPL10A;BLMH | 4 | 1;2;3;4 | 0;1;2;2 |
| RPL24 | RPL24;NEDD8;PINX1;CUL5 | 4 | 1;2;3;4 | 0;1;1;2 |
| RPL36AL | RPL36AL;ESR1;RPL15;TRRAP;NRIP1 | 5 | 1;2;3;4;5 | 0;1;2;2;2 |
| RPLP2 | RPLP2;ADRBK1;MAP1LC3B;SQSTM1;CAV1 | 5 | 1;2;3;4;5 | 0;1;1;3;2 |
| RPS2 | RPS2;NEDD8;GABARAP;SQSTM1 | 4 | 1;2;3;4 | 0;1;1;3 |
| RPS9 | RPS9;MAP3K1;MYH10;KRT10 | 4 | 1;2;3;4 | 0;1;2;1 |
| RSU1 | RSU1;RAF1;MAPK7;CDC25A;STUB1 | 5 | 1;2;3;4;5 | 0;1;2;2;2 |
| RWDD1 | RWDD1;NDRG1;AP2M1;FASN;LY9 | 5 | 1;2;3;4;5 | 0;1;2;2;3 |
| SCARB2 | SCARB2;PLCG1;MAP4;NTRK1;GRIN2A | 5 | 1;2;3;4;5 | 0;1;2;2;2 |
| SCN4A | SCN4A;SLC9A3R1;CTNNB1;CDH1 | 4 | 1;2;3;4 | 0;1;2;3 |
| SEC24A | SEC24A;GABARAPL1;EEF2;ANXA2;CCT8 | 5 | 1;2;3;4;5 | 0;1;2;2;2 |
| SELPLG | SELPLG;SYK;GRB2;KRT18;PIK3R2 | 5 | 1;2;3;4;5 | 0;1;2;3;2 |
| SHCBP1 | SHCBP1;SFN;SH3BP4;MARK3;REPS2 | 5 | 1;2;3;4;5 | 0;1;2;2;2 |
| SHOX2 | SHOX2;TRIM29;VIM;RIPK3 | 4 | 1;2;3;4 | 0;1;2;3 |
| SLC25A6 | SLC25A6;EIF2C1;RPL19;SIRT4 | 4 | 1;2;3;4 | 0;1;2;1 |
| SLC27A5 | SLC27A5;HSF4;MAPK3;JUND;TOP2B | 5 | 1;2;3;4;5 | 0;1;2;3;3 |
| SMARCAD1 | SMARCAD1;SNW1;UBE2N;BUB1B | 4 | 1;2;3;4 | 0;1;2;2 |
| SP4 | SP4;SP3;SP1;NFKB1;IKBKG;SMARCC2 | 6 | 1;2;3;4;5;6 | 0;1;1;3;4;3 |
| SSR1 | SSR1;EIF2C1;RPL35;RPS23;RPL19;TCP1 | 6 | 1;2;3;4;5;6 | 0;1;2;2;2;2 |
| STMN2 | STMN2;MAPK3;ELK1;HSF4 | 4 | 1;2;3;4 | 0;1;2;2 |
| SYNE1 | SYNE1;DISC1;ITSN1;MACF1 | 4 | 1;2;3;4 | 0;1;2;2 |
| SYNPO2 | SYNPO2;YWHAZ;CHEK1;EPB41L2 | 4 | 1;2;3;4 | 0;1;2;2 |
| SYP | SYP;EPOR;PLCG1;SOS2 | 4 | 1;2;3;4 | 0;1;2;3 |
| TARS | TARS;NEDD8;EEF2;RPL23;MAGED1 | 5 | 1;2;3;4;5 | 0;1;2;2;2 |
| TEC | TEC;DOK1;ITGB3;EPS8 | 4 | 1;2;3;4 | 0;1;2;3 |
| TFAP2A | TFAP2A;NPM1;SH3BP4;CITED2 | 4 | 1;2;3;4 | 0;1;1;1 |
| TGFBR3 | TGFBR3;SDC2;FN1;SDC1;ADAMTS4;C1QA | 6 | 1;2;3;4;5;6 | 0;1;2;2;3;3 |
| TIMELESS | TIMELESS;CHEK1;SFN;PAK4;PER1 | 5 | 1;2;3;4;5 | 0;1;2;3;1 |
| TLR8 | TLR8;BTK;IRAK1;SYK;IKBKB;MAP3K1 | 6 | 1;2;3;4;5;6 | 0;1;2;2;3;5 |
| TNFRSF10B | TNFRSF10B;TRADD;SLC25A6;IPO7;CAV1 | 5 | 1;2;3;4;5 | 0;1;2;2;2 |
| TREM1 | TREM1;TYROBP;SYK;CD22;TREM2;FCGR2A | 6 | 1;2;3;4;5;6 | 0;1;2;3;2;3 |
| TRIP10 | TRIP10;DAAM1;DNM2;WBP4 | 4 | 1;2;3;4 | 0;1;1;3 |
| TRRAP | TRRAP;ESR1;RPL15;RPL12 | 4 | 1;2;3;4 | 0;1;2;2 |
| TSC2 | TSC2;CAV1;CDH1;DNM1 | 4 | 1;2;3;4 | 0;1;2;2 |
| TTBK2 | TTBK2;GABARAPL2;SQSTM1;EEF2;SLC25A5 | 5 | 1;2;3;4;5 | 0;1;2;2;2 |
| VCL | VCL;TLN1;ITGB3;DOK1;PSME1 | 5 | 1;2;3;4;5 | 0;1;2;3;1 |
| VIM | VIM;MAP3K1;RIPK3 | 3 | 1;2;3 | 0;1;1 |
| WDFY3 | WDFY3;MAP1LC3B;EEF2;SPTBN1 | 4 | 1;2;3;4 | 0;1;2;2 |
| WFDC2 | WFDC2;PTN;BCCIP;PTPRS | 4 | 1;2;3;4 | 0;1;2;2 |
| WSB1 | WSB1;HIPK2;CREBBP;STAT1;MAFG;DAXX | 6 | 1;2;3;4;5;6 | 0;1;2;3;3;2 |
| XRCC2 | XRCC2;RAD51;BCCIP;BRCA1;CHEK1 | 5 | 1;2;3;4;5 | 0;1;2;2;2 |
| ZNF35 | ZNF35;CREB1;SOX9;VIM;NR5A1 | 5 | 1;2;3;4;5 | 0;1;2;2;3 |
