# Supplementary material for: A Systems Biology Comparison of Ovarian Cancers Implicates Putative Somatic Driver Mutations through Protein-Protein Interaction Models
Source: PLoS One. 2016 Oct 27;11(10):e0163353. doi: 10.1371/journal.pone.0163353 (PMC5082879; doi:10.1371/journal.pone.0163353)
Supplement: S2 Table — (DOCX) [file pone.0163353.s012.docx]

**S2 Table. The common significant hub proteins**

| **Hub protein** | **Number of interacting proteins** | **P-value (GSE9891)** | **P-value (GSE17308)** |
| --- | --- | --- | --- |
| HNRPDL | 10 | 0 | 0.004 |
| SMARCD2 | 7 | 0 | 0.05 |
| THBS1 | 35 | 0 | 0.04 |
| PPID | 13 | 0 | 0.031 |
| DCN | 28 | 0 | 0.042 |
| UPF2 | 19 | 0.001 | 0.031 |
| WWOX | 19 | 0.001 | 0.05 |
| SNRPC | 10 | 0.002 | 0.022 |
| APEX1 | 59 | 0.002 | 0 |
| ITGB5 | 22 | 0.002 | 0.028 |
| NID2 | 9 | 0.002 | 0.001 |
| CSRP1 | 47 | 0.003 | 0.05 |
| TIAM1 | 24 | 0.004 | 0.021 |
| MAP1LC3A | 234 | 0.005 | 0.029 |
| XPOT | 14 | 0.005 | 0.003 |
| CREBBP | 212 | 0.005 | 0.032 |
| CDT1 | 35 | 0.005 | 0.003 |
| EFNB1 | 12 | 0.005 | 0.05 |
| RPL19 | 10 | 0.006 | 0.048 |
| RANBP2 | 27 | 0.007 | 0.004 |
| DAG1 | 23 | 0.007 | 0.015 |
| DDX5 | 53 | 0.008 | 0.03 |
| PPP2R5C | 13 | 0.01 | 0.03 |
| WWP2 | 18 | 0.01 | 0.045 |
| RALA | 16 | 0.01 | 0.003 |
| ELK1 | 22 | 0.01 | 0.01 |
| MAP4 | 14 | 0.01 | 0.05 |
| RPS6KA3 | 20 | 0.01 | 0 |
| INADL | 28 | 0.01 | 0.017 |
| SCAMP1 | 9 | 0.01 | 0.007 |
| GRIN2D | 27 | 0.01 | 0.036 |
| NCOR1 | 88 | 0.01 | 0.033 |
| EEF2 | 22 | 0.01 | 0 |
| AKAP9 | 25 | 0.01 | 0.04 |
|  |  |  |  |
