# Supplementary material for: A Systems Biology Comparison of Ovarian Cancers Implicates Putative Somatic Driver Mutations through Protein-Protein Interaction Models
Source: PLoS One. 2016 Oct 27;11(10):e0163353. doi: 10.1371/journal.pone.0163353 (PMC5082879; doi:10.1371/journal.pone.0163353)
Supplement: S3 Table — (DOCX) [file pone.0163353.s013.docx]

**S3 Table. The performance of classification using different type molecular signature as features**

| **Type of signatures** | **Type of classify** | **Data** | **Sensitivity** | **Sen.Sd** | **Specificity** | **Spec.sd** | **Accuracy** | **Accu.sd** | **AUC** |
| --- | --- | --- | --- | --- | --- | --- | --- | --- | --- |
| **Differentially expressed genes** | In cohort | GSE17308 | 1 | 0 | 1 | 0 | 1 | 0 | 1 |
|  |  | GSE9891 | 0.994 | 0.03 | 0.991 | 0.012 | 0.992 | 0.011 | 0.997 |
|  | Between cohorts | GSE17308 | 0.95 | 0.153 | 0.99 | 0.036 | 0.981 | 0.051 | 0.998 |
|  |  | GSE9891 | 0.989 | 0.061 | 0.987 | 0.013 | 0.987 | 0.013 | 0.992 |
| **Subnetworks** | In cohort | GSE17308 | 1 | 0 | 1 | 0 | 1 | 0 | 1 |
|  |  | GSE9891 | 1 | 0 | 0.991 | 0.012 | 0.993 | 0.011 | 1 |
|  | Between cohorts | GSE17308 | 0.967 | 0.183 | 0.995 | 0.026 | 0.989 | 0.045 | 0.993 |
|  |  | GSE9891 | 0.994 | 0.03 | 0.989 | 0.013 | 0.99 | 0.013 | 0.993 |
| **Hub proteins** | In cohort | GSE17308 | 0.783 | 0.284 | 1 | 0 | 0.952 | 0.063 | 0.998 |
|  |  | GSE9891 | 0.983 | 0.051 | 0.991 | 0.012 | 0.99 | 0.014 | 0.997 |
|  | Between cohorts | GSE17308 | 0.583 | 0.396 | 0.981 | 0.049 | 0.893 | 0.09 | 0.974 |
|  |  | GSE9891 | 0.989 | 0.042 | 0.991 | 0.012 | 0.991 | 0.013 | 0.998 |
